# Design and *in silico* evaluation of Thiazole–Isoxazole hybrids as PPARγ-Targeted antidiabetic agents

**DOI:** 10.3389/fbinf.2026.1873262

**Published:** 2026-08-11

**Authors:** Abhirami PV, Gupta Dheeraj Rajesh, N. V. L. Sirisha Mulukuri, Ranjitha A, Niyas Rehman, Shiv Basant Kumar, Dileep Kumar, Pankaj Kumar

**Affiliations:** 1 Department of Pharmaceutical Chemistry, NGSM Institute of Pharmaceutical Sciences (NGSMIPS), Nitte (Deemed to be University), Mangalore, India; 2 Department of Pharmacognosy, Nitte College of Pharmaceutical Sciences (NCOPS), Nitte (Deemed to be University), Bangalore, India; 3 Centre for Integrative Omics Data Science, Yenepoya (Deemed to be University), Mangalore, Karnataka, India; 4 Australian Plastic Research and Innovation Lab, Global Innovative Centre for Advanced Nanomaterials (GICAN), College of Engineering, Science and Environment (CESE), The University of Newcastle, Callaghan, NSW, Australia; 5 Department of Pharmaceutical Chemistry, Manipal College of Pharmaceutical Sciences, Manipal Academy of Higher Education, Manipal, Karnataka, India

**Keywords:** anti-diabetic, isoxazole, molecular docking, molecular dynamic simulation, peroxisome proliferator-activated receptor gamma, thiazole

## Abstract

**Introduction:**

Diabetes mellitus is a chronic metabolic disorder characterised by persistent hyperglycemia resulting from impaired insulin secretion, action, or both. Prolonged hyperglycemia disrupts metabolic homeostasis and increases the risk of complications, including cardiovascular disease, neuropathy, nephropathy, and retinopathy. Current therapies target multiple pathways but are limited by reduced efficacy and adverse effects. Peroxisome proliferator-activated receptor gamma (PPARγ) is a key regulator of glucose and lipid metabolism and an important therapeutic target. Thiazole and isoxazole scaffolds possess antidiabetic potential, and their hybridisation may improve efficacy and safety.

**Objective:**

To design and evaluate a novel series of thiazole-linked isoxazole derivatives targeting the alternate ligand-binding domain of PPARγ for potential antidiabetic activity.

**Result:**

The designed compounds (C1–C210) were docked into PPARγ (Protein Data Bank: 5 GTN), yielding nine candidates with binding affinities of −11.11 to −9.97 kcal/mol. Reference ligands included pioglitazone, Q-35, and S-35, with C139 showing the highest affinity. Molecular dynamics simulations (200 ns) confirmed stability. Advanced Trajectory analyses indicate favourable conformational sampling, correlated residue motions, and a relatively confined conformational space. *In silico* drug-likeness profiling further supported their activity profile.

**Conclusion:**

In this computational study, the thiazole-linked isoxazole compound C139 demonstrated probable antidiabetic activity against PPARγ. However, these findings remain predictive and require further validation through comprehensive *in vitro* and *in vivo* studies.

## Graphical Abstract

**Figure:**
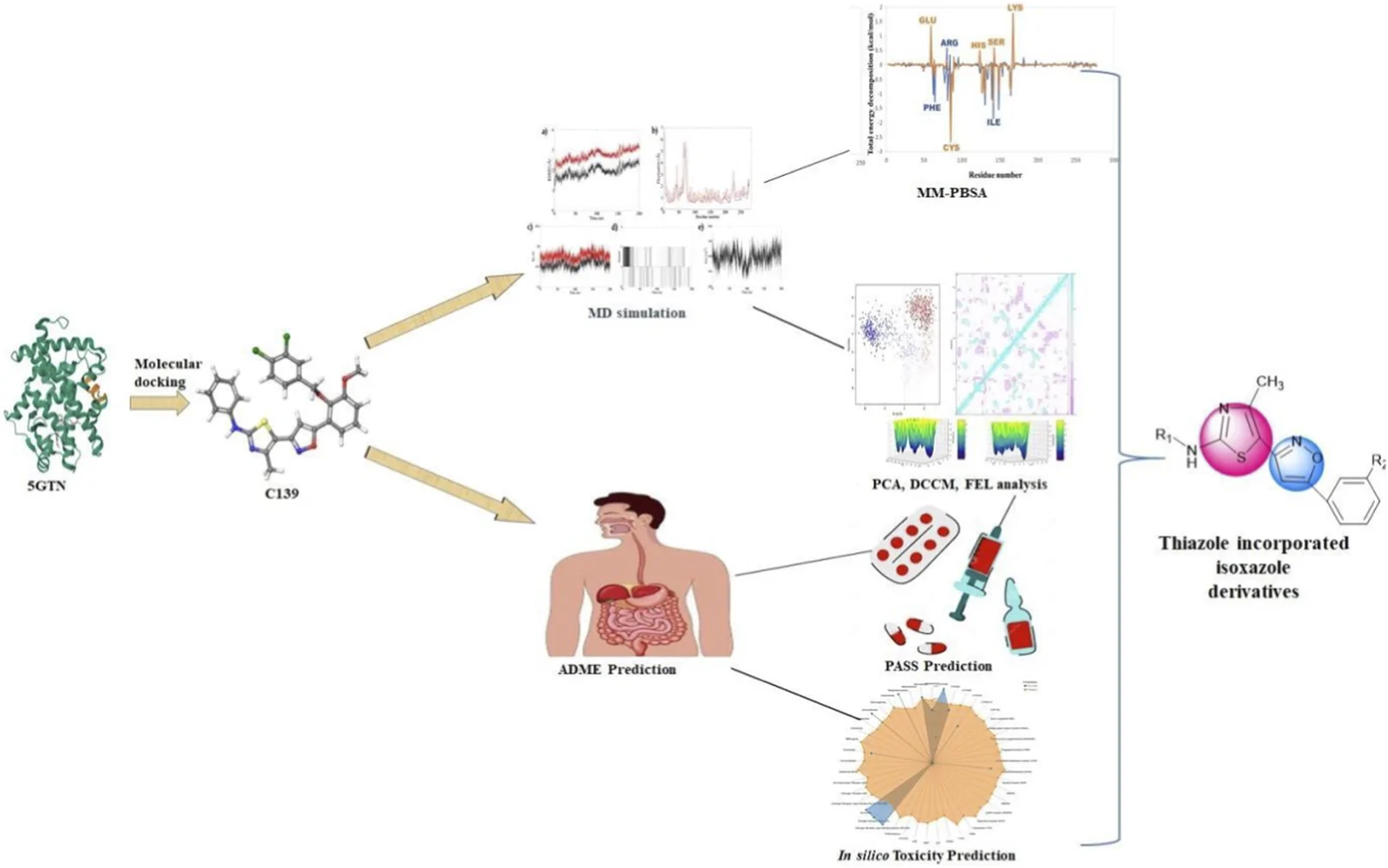

## 1 Introduction

**FIGURE 1:**
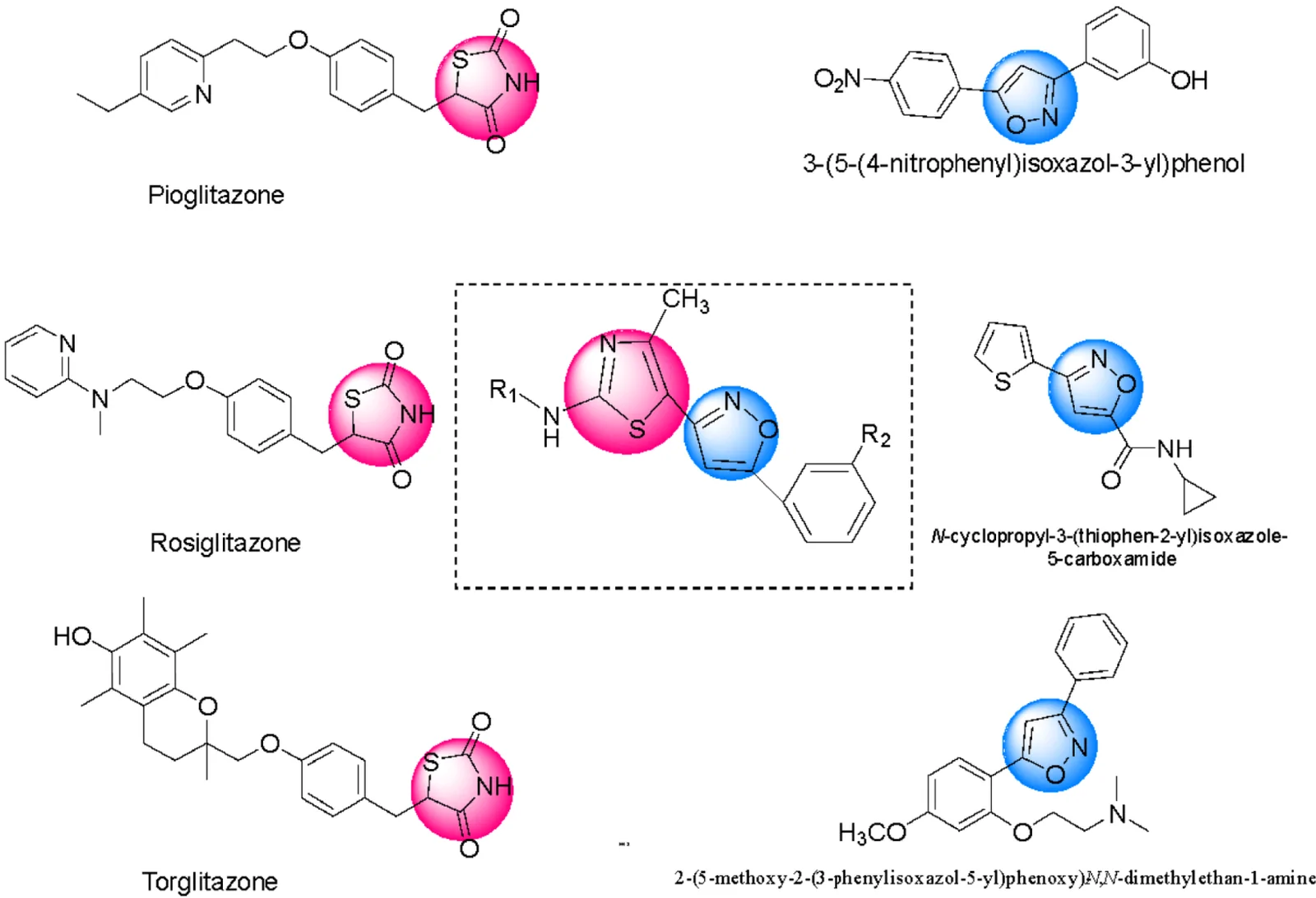
Structure of the designed thiazole-incorporated isoxazole derivatives.

Diabetes mellitus (DM) is a complex metabolic disease characterised by persistently elevated blood sugar levels due to impaired metabolism of carbohydrates, fats, and proteins. Globally, an estimated 422 million individuals are affected, mainly the residents of low- and middle-income countries, responsible for approximately 1.5 million deaths annually, and both its prevalence and incidence have shown a steady upward trend over recent decades (Zuhara, 2024; Saeedi et al., 2019). People across Asia have a higher predisposition to get Type 2 Diabetes Mellitus (T2DM) at younger ages as compared to other regions of the world. Notably, India is now recognized as a global hotspot for diabetes, with an estimated 62 million affected individuals (Kaveeshwar and Cornwall, 2014; Unnikrishnan et al., 2016). It is linked with different organs’ dysfunction, damage and failure, including the kidneys, retina, heart, nervous system, and vascular system. Current therapeutic approaches for T2DM involve a range of pharmacological agents targeting distinct physiological processes. These include drugs aimed at reducing hepatic glucose production (Metformin), enhancing insulin secretion through pancreatic β-cells (Sulfonylureas, Meglitinides, Glucagon-Like Peptide-1 (GLP-1) agonists, dipeptidyl peptidase-4 (DPP-4) inhibitors, suppressing glucagon release from pancreatic α-cells (GLP-1 agonists, DPP-4 inhibitors), refining insulin sensitivity in muscle and adipose tissue via PPARγ activation (Thiazolidinediones), inhibiting renal glucose reabsorption through the kidneys Sodium-Glucose Co-Transporter 2 (SGLT2)inhibitors), and delaying carbohydrate absorption as well as potentiating incretin effects in the gut (α-glucosidase inhibitors, GLP-1-based therapies) (Alam et al., 2014; Khan, 2024). Despite the availability of existing therapies, optimal management of T2DM remains challenging due to suboptimal glycemic control, medication-associated adverse effects, and increased risks of cardiovascular disorders, stroke, microvascular complications, impaired immune response, infections, and delayed wound healing, highlighting the urgent need for the design and development of novel, safe, and effective antidiabetic molecules that can lead to improved therapeutic outcomes (Zakir et al., 2023; Dowarah and Singh, 2020; Mishra et al., 2021). PPARγ plays a crucial role in regulating protein expression in carbohydrate and lipid metabolism and is a key molecular target for diabetes therapy (Khasanova et al., 2022). This nuclear receptor modulates insulin sensitivity and is the pharmacological target of Thiazolidinedione (TZDs), which enhance insulin action and glucose utilization (Jang et al., 2023). Due to its critical role in metabolic regulation, the alternate ligand-binding domain, located in the Ω loop amid helices H2 and H3 of PPARγ, was selected as the primary target for the present investigation (Jang et al., 2017). The alternate binding site offers a distinct mechanism of PPARγ modulation by stabilizing the Ω-loop and inhibiting Cyclin-Dependent Kinase-5 (CDK5)-mediated phosphorylation, a process closely linked to insulin sensitization. Unlike classical agonists, alternate-site ligands may provide therapeutic benefits with reduced receptor overactivation and fewer adverse effects. Thiazoles, particularly 1,3-thiazole—are a heterocyclic five-membered ring having nitrogen and sulfur heteroatoms—and are important structural motifs in medicinal chemistry. This scaffold occurs in numerous bioactive molecules and serves as a valuable pharmacophore for the design of novel therapeutics. Substituted thiazoles have been reported to exhibit diverse pharmacological activities, including antibacterial (Khalil et al., 2009), anticonvulsant (Siddiqui et al., 2020), anticancer (Farghaly et al., 2020), antitubercular, antiproliferative (Kasetti et al., 2021), and antidiabetic (Rajesh et al., 2023) effects. Clinically approved antidiabetic agents with a thiazole core include pioglitazone, rosiglitazone, and troglitazone (Khan et al., 2002). This moiety contributes to high-affinity binding and activation of PPARγ through favourable interactions within the ligand-binding domain that help to stabilize the receptor’s active conformation and facilitate coactivator recruitment. Consequently, the thiazole scaffold plays a significant role in PPARγ-mediated insulin sensitization and antidiabetic activity. Likewise, the isoxazole ring—a five-membered heterocycle with nitrogen and oxygen atoms at positions one and two—exhibits broad pharmacological relevance. Isoxazole derivatives readily interact with diverse enzymes and receptors, and their incorporation into drug scaffolds can modulate activities such as antiviral, anticancer, immunomodulatory, anti-inflammatory, analgesic (Sysak and Obmińska-Mrukowicz, 2017), antioxidant (Hawash et al., 2022), antibacterial, and antidiabetic effects. Notable isoxazole-based antidiabetic scaffolds include 3-(5-(4-nitrophenyl)isoxazol-3-yl)phenol (Joseph and George, 2016) and N-cyclopropyl-3-(thiophen-2-yl)isoxazole-5-carboxamide (Shilpa et al., 2023). Isoxazole derivatives have been reported to modulate PPARγ activity through favorable interactions within its ligand-binding domain, thereby enhancing glucose uptake and improving insulin sensitivity. Furthermore, the isoxazole ring imparts structural rigidity and hydrogen-bonding capability, which facilitates effective receptor binding and contributes to improved pharmacological activity. Although the antidiabetic potential of thiazole- and isoxazole-based scaffolds as individual pharmacophores has been reported, molecules that integrate both moieties within a single framework are scarce. With this rationale, two different pharmacophores—thiazole and isoxazole—were conjugated to form a hybrid scaffold. The hybridisation strategy was intended to combine the favourable pharmacological attributes of both heterocyclic moieties while introducing key pharmacophoric features, including hydrogen-bond donor and acceptor sites, hydrophobic regions, aromatic functionalities, and potential electronic interactions, all of which are essential for effective receptor binding and biological activity. In addition, scaffold hybridisation may improve pharmacokinetic and ADME (Absorption, Distribution, Metabolism, and Excretion) properties. Guided by this design strategy, a series of derivatives was subsequently developed through the introduction of diverse substituents onto the core scaffold to explore different derivatives and explore biological potential The design strategy was informed by previously reported analogues containing either thiazole or isoxazole moieties with established antidiabetic activity, as illustrated in Figure 1. Molecular docking studies were conducted against the identified PPARγ receptor to evaluate the interaction capabilities of the designed ligands. Lead candidates were further evaluated for stability with the receptor using a comprehensive computational method that includes molecular dynamics (MD) simulation, molecular mechanics Poisson–Boltzmann surface area (MMPBSA) calculations, principal component analysis (PCA), free energy landscape (FEL) mapping, and dynamic cross-correlation matrix (DCCM) analysis. The functional groups and spatial arrangements of the compounds that result in the desired biological activity were predicted using a pharmacophore model. Additionally, *in silico* predictions of bioactivity and toxicity were carried out using the Predicting Activity Spectra for Substances (PASS) online and ProTox 3.0, respectively, and were compared with co-crystals and standard drugs. Computational approaches, such as docking and dynamics, have now become integral to modern drug discovery. These techniques enable precise interactions between candidate ligands and target proteins, providing critical insights into the stability, specificity, and potential efficacy of drug–target complexes. By simulating ligand binding and monitoring dynamic conformational changes, such analyses contribute significantly to lead optimisation and accelerate the development of novel therapeutic agents for unmet clinical needs (Alonso et al., 2006).

## 2 Materials and methods

All *in silico* evaluations were conducted using Maestro (version 14.0.136, Schrödinger, LLC) and GROMACS on a Dell 27-inch workstation configured with an Intel Core i7-7,700 processor (3.60 GHz, eight cores), 8 GB RAM, and a 1 TB hard drive, operating on a Linux ×86_64 platform. Schrödinger’s Maestro software is widely recognised for its advanced capabilities in molecular docking, whereas GROMACS is extensively utilised for MD in drug development.

### 2.1 Design and preparation of ligands

The designed compounds (C1–C210), generated by introducing structurally diverse electron-donating, electron-withdrawing, and neutral substituents at different aromatic positions to modulate their steric, electronic, and hydrophobic interactions with PPARγ, were evaluated for their predictive antidiabetic potential through comprehensive *in silico* studies (Supplementary Files S1, S2). Two-dimensional structures of all ligands were generated in ChemDraw and subsequently converted to three-dimensional conformations using LigPrep. The energetically stable conformations were chosen for further analyses (Dheeraj et al., 2023).

### 2.2 Preparation and structure validation of the receptor

The crystal structure of the human PPARγ ligand-binding domain (PDB ID: 5 GTN) was downloaded from the RCSB repository (www.rcsb.org) and processed. The protein exists as a homodimer comprising chains A and B; however, only Chain A was selected for subsequent computational analyses. It was prepared using a preparation wizard that involved refinement, optimisation, and the application of structural constraints, and validated using Ramachandran plots generated by the same module. These plots categorise amino acid residues into three categories: most favoured, additionally allowed, and outlier regions. Residues were distributed across these regions, with a notable clustering in the right-handed α-helix domain. Additional residues were also located within β-sheet and collagen triple-helix conformations. Above 90% of the residues were observed in the prominent regions of the Ramachandran plot, indicating that the protein structure has high overall quality (Hollingsworth and Karplus, 2010).

### 2.3 Molecular docking

Molecular docking is a widely used computational approach in structure-based drug design that predicts the preferred binding orientation of a ligand within the active site of a target protein and provides insights into the molecular interactions governing ligand–receptor recognition. For the present study, the crystal structure of PPARγ (PDB ID: 5 GTN) was retrieved from the PDB. Although the receptor exists as a homodimer, only Chain A was selected for molecular docking because it contains the complete ligand-binding domain along with the co-crystallised ligand within a well-defined active site. Binding site grids were generated using Schrödinger’s receptor grid generation module. Subsequently, the designed ligands were then docked into the active site using the Glide Extra Precision (XP) docking protocol. The binding affinities of the designed compounds were evaluated based on their docking scores (kcal/mol) and compared with those of the standard antidiabetic drug pioglitazone, the co-crystallized PPARγ agonist Q-35 [2-[4-[5-[(1 R)-1-(3,5-dimethoxyphenyl)carbamoyl-(phenylmethyl)carbamoyl]oxypropyl]-1,2-oxazol yl]phenoxy]-2-methylpropanoic acid], and the co-crystallized PPARγ antagonist S-35 [2-[4-[5-[(1 S)-1-(3,5-dimethoxyphenyl)carbamoyl-(phenylmethyl)carbamoyl]oxypropyl]-1,2-oxazol-yl] phenoxy]-2-methylpropanoic acid] (Rajesh et al., 2025). These reference ligands were selected to provide established pharmacological and structural benchmarks for evaluating the binding characteristics of the designed compounds within the PPARγ ligand-binding domain Molecular docking was validated by redocking the co-crystallised ligand into the active site of PPARγ (PDB ID: 5 GTN) using the same Glide docking parameters and the Root Mean Square Deviation (RMSD)was calculated.

### 2.4 Molecular dynamic simulation

To evaluate the binding stability of the top-docked thiazole-fused isoxazole derivative complexed with the human PPARγ protein (PDB ID: 5 GTN), a 200 ns all-atom MD simulation was carried out using GROMACS version 2021.6. Initially, the protein was stabilised, and its topology was generated with the pdb2gmx module using the CHARMM36 force field and Swiss-Param. The complex was placed in a dodecahedral simulation box with a 1 nm buffer from the edge and solvated with the three-point Transferable Intermolecular Potential (TIP3P) water model. It was neutralised with 0.1 M sodium and chloride ions and kept electrostatically balanced. The complex energy minimum was performed with the steepest descent algorithm. The system was equilibrated for 100 ps under constant-volume (NVT) and constant-pressure (NPT) conditions, respectively. The system temperature was maintained at 300 K using the Berendsen Barostat, while the pressure was set to 1 bar using the C-rescale barostat. The Particle Mesh Ewald (PME) technique, with a 1 nm cutoff, was used for long-range electrostatic and van der Waals interactions, while bond lengths were constrained using the LINCS algorithm. The simulated trajectories were calculated using GROMACS built-in tools to evaluate critical properties, including RMSD, Root Mean Square Fluctuation (RMSF), Radius of Gyration (RoG), Hydrogen-bond (H-bond) count, and Solvent-Accessible Surface Area ***(*** SASA). Discovery Studio Visualizer (DSV) was used to visualise the initial and final structures of the protein–ligand complex (Rajendran et al., 2023; Berendsen et al., 1995).

### 2.5 Principal component analyses

This statistical technique defines the main patterns of variation in a dataset by evaluating its covariance or correlation matrix. In MD simulations, atomic coordinates are used to describe the principal degrees of freedom (DOF) associated with protein motion. In this study, PCA was applied to reveal the protein’s major conformational dynamics. The covariance matrix and corresponding eigenvalues were initially computed using the Bio3D package (version 3.3) in RStudio. Diagonalization of the covariance matrix yielded to the eigenvectors, which represent the principal components of motion. To capture the relevant atomic displacements sampled during the simulation, the gmx covar tool from the GROMACS suite was utilized. Graphical representations of the principal motions were subsequently generated using Mathematica (version 13.3) (Rajesh et al., 2024a).

### 2.6 Dynamic cross-correlation map analysis

The DCCM was employed to identify potential functional domains within the protein structure. This method constructs a three-dimensional matrix that captures the time-dependent correlated motions of amino acid residues, based on Cα atom fluctuations across all protein–ligand complexes. MD simulation trajectories were used to generate DCCM, enabling investigation of correlated and anticorrelated movements between residues. The Bio3D package was used to compute and visualize these maps, thereby facilitating the interpretation of dynamic inter-residue interactions and the identification of domain-level motion within the protein (Rajesh et al., 2024a).

### 2.7 Free energy landscape analyses

This analysis, based on PCA results, was performed to identify the conformational frame corresponding to the lowest Gibbs free energy. Key parameters, including RMSF, RoG, and Gibbs free energy, were computed as part of this analysis. Two-dimensional and three-dimensional representations of the FEL were generated using Mathematica (version 12), providing insights into the conformational states critical to the protein’s structure–function relationship, which is governed by both enthalpic and entropic contributions. The g_sham tool from the GROMACS suite was used to calculate the free FEL from the Cα atom trajectories obtained from the three simulated systems (Rajendran et al., 2023).

### 2.8 Molecular mechanics Poisson-Boltzmann surface area analysis

This module is used to estimate the binding free energy and break down the individual energy components of the ligand–5GTN complex. The main parameters evaluated Van der Waals and electrostatic interactions, overall gas-phase energy, total solvation energy, overall binding free energy, along with the polar and nonpolar components of solvation. Per-residue energy decomposition was further conducted to identify nonpolar and attractive energy contributions at the residue level. The MM-PBSA calculation was conducted over 1,000 frames extracted at 10-frame intervals from the 200 ns MD trajectory. Poisson–Boltzmann (PB) calculations were performed using the PBSA internal solver in the sander module as part of the MMPBSA. py analytical framework (Valdés-Tresanco et al., 2021; Tharamelveliyil Rajendran et al., 2024).

### 2.9 Physicochemical properties and absorption, distribution, metabolism, excretion

The evaluation of *in silico* ADME properties has become a crucial approach for assessing a compound’s potential as a drug candidate. The drug-likeness and pharmacokinetic properties of the designed ligands were assessed using the QikProp module (Schrödinger, LLC). The parameters evaluated using this module included molecular weight, the number of hydrogen bond donors and acceptors, dipole moment, and the predicted octanol/water partition coefficient (log P). Based on these parameters, Lipinski’s Rule of Five was applied to determine the drug-likeness and potential oral bioavailability of the designed ligands.

### 2.10 Generation of pharmacophore modeling

The models employed in this study were prepared by the Phase module (Schrödinger, LLC). A receptor-based pharmacophore approach was utilized to characterize three-dimensional spatial arrangement of molecular features essential for receptor binding and potential bioactivity (Rajendran et al., 2023).

### 2.11 Predicting Activity Spectra for Substances

The computational tool PASS employed to evaluate the antidiabetic potential of the designed ligand candidates. Canonical Simplified Molecular Input Line Entry System (SMILES) representations of the compounds were submitted to the PASS server, which generated probability estimates for pharmacological inactivity (Pi) and pharmacological activity (Pa). Pa value represents the likelihood that a compound exhibits a specific biological activity, with higher Pa values indicating a greater probability that the ligand possesses the predicted pharmacological effect (Dheeraj et al., 2023).

### 2.12 Toxicity prediction by ProTox 3.0

ProTox 3.0, an accessible internet-based platform, was utilized to predict the toxicity profiles of the ligands. This computational tool employs advanced algorithms to estimate various toxicity endpoints, including toxicity classes and median lethal dose (LD_50_) values expressed in mg/kg of body weight, which denote the dose required to cause mortality in 50% of an experimental group. The toxicity assessment was performed by entering the canonical SMILES representations of each compound into the ProTox 3.0 interface (Rajesh et al., 2024b; Banerjee et al., 2024).

## 3 Results and discussion

### 3.1 Molecular docking

**FIGURE 2:**
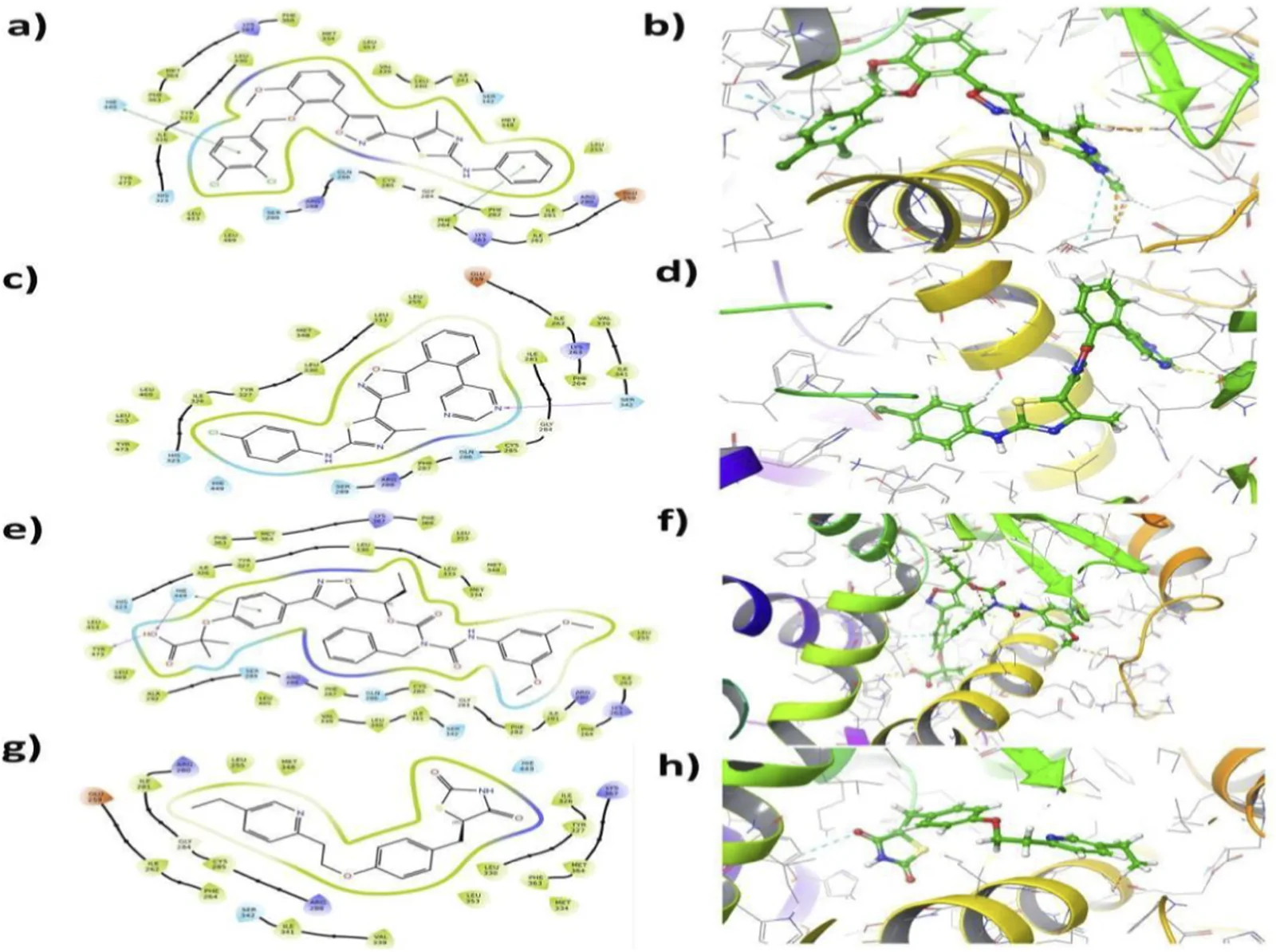
2D and 3D interaction with 5 GTN **(a,b)** C139 **(c,d)** C184 **(e,f)** Q-35 **(g,h)** Pioglitazone.

The designed compounds (C1–C210) were docked into the ligand-binding site of the PPARγ receptor Protein Data Bank (PDB) ID: 5 GTN, and their docking scores and binding modes were analyzed (Supplementary File 2). Docking was employed as an initial screening tool to assess the potential of the compounds to interact with the receptor and to prioritize candidates for further investigation. Based on a combination of docking scores and binding interactions relative to the reference ligand, the top nine compounds were selected for subsequent evaluation of their drug-likeness, pharmacokinetic properties, and predicted antidiabetic potential. Selected compounds’ docking scores ranged from −11.11 to −9.97 kcal/mol, whereas the reference drug pioglitazone (−7.63 kcal/mol), Q-35 (−10.33 kcal/mol), and the S-35 (antagonist, enantiomer of Q-35) were summarized in Table 1. Compound C139 and C184 exhibited the most favourable binding affinities. Compound C139 exhibited the highest docking score of −11.11 kcal/mol, forming a π–π stacking interaction with PHE264, which highlights a strong aromatic interaction within the PPARγ binding pocket. Compound C184 formed a hydrogen bond between its nitrogen atom and SER342, with a bond distance of 3.65 Å, indicating a specific and stabilizing polar interaction. Whereas Q-35 engaged the receptor through two hydrogen bonds with residues HIE449 and TYR473, and π–π stacking bonding. In contrast, the reference ligand pioglitazone did not form hydrogen bonds but interacted with receptor residues via π–π stacking, π–cation interactions, halogen bonding, and a variety of hydrophobic, polar, and electrostatic interactions. Whereas Docking protocol validation with RMSD was 0.22Å. Detailed interaction profiles of the top nine compounds, along with pioglitazone and the Q-35, are provided in (Supplementary File S2). Representative ligand–receptor interactions for C139, C184, pioglitazone, and the Q-35 are illustrated in Figure 2.

**TABLE 1:** The structure and glide score of the top nine compounds docked to 5 GTN receptor.

Sl No	Compound code	Docking score (kcal/mol)	Structures
1	C139	−11.11	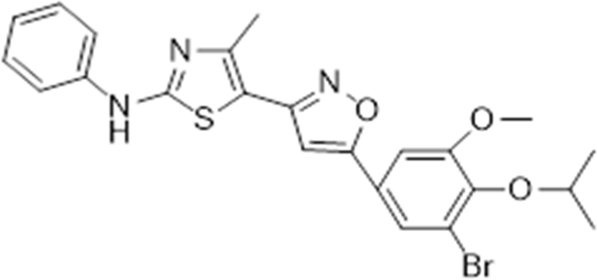
2	C184	−10.45	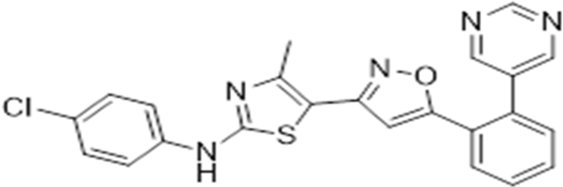
3	C107	−10.27	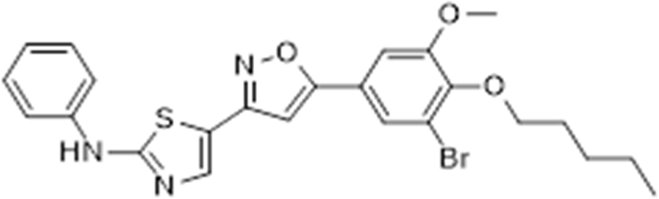
4	C131	−10.20	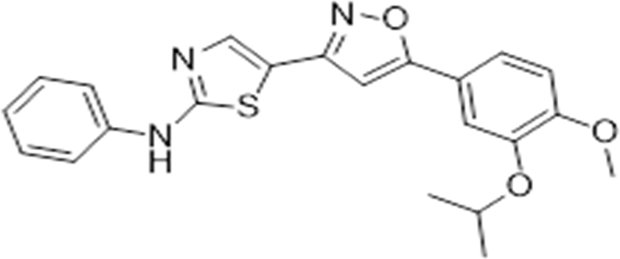
5	C167	−10.17	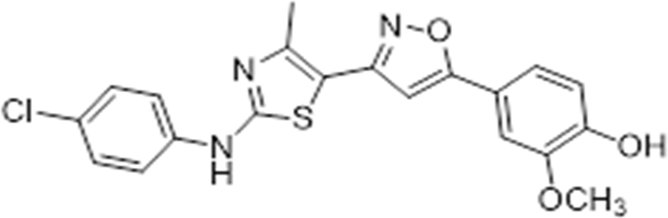
6	C113	−10.16	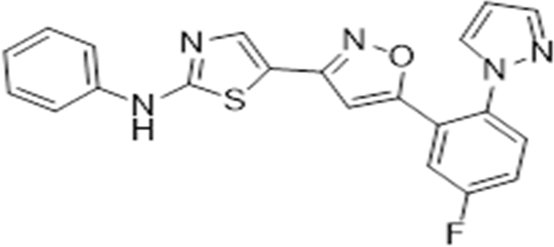
7	C202	−10.01	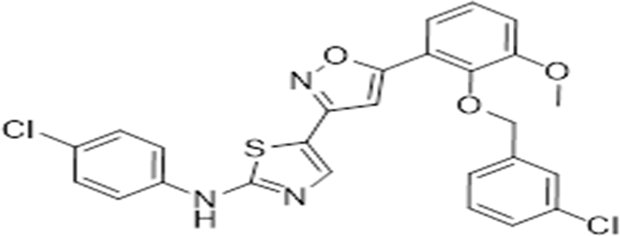
8	C37	−9.99	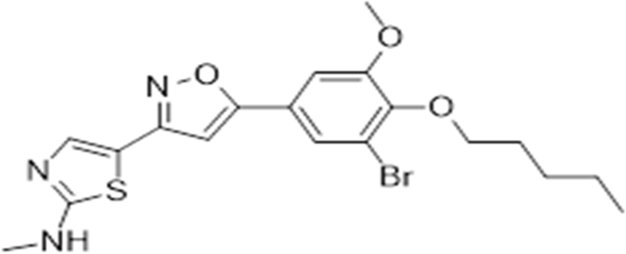
9	C29	−9.97	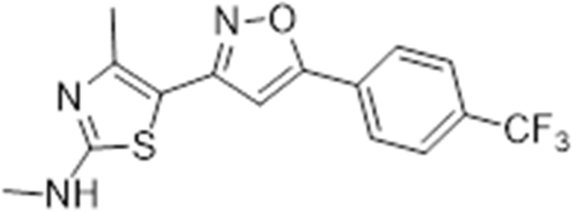
10	(Co-crystal) (Q-35) R-35	−10.33	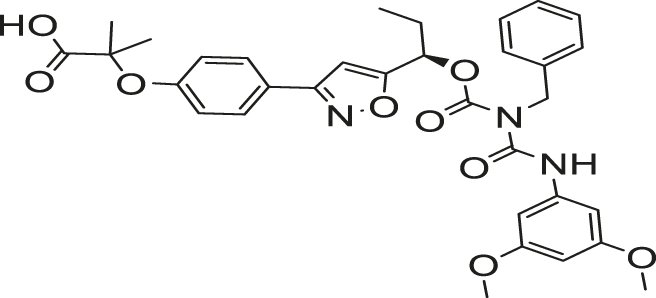
11	S-35	−3.048	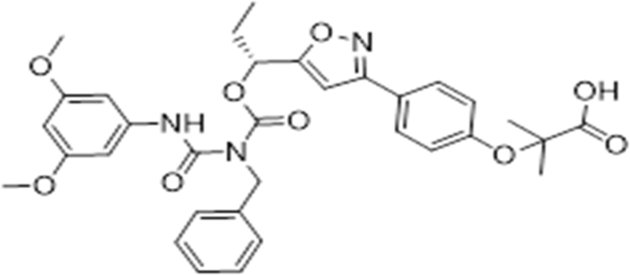
12	Pioglitazone (std)	−7.63	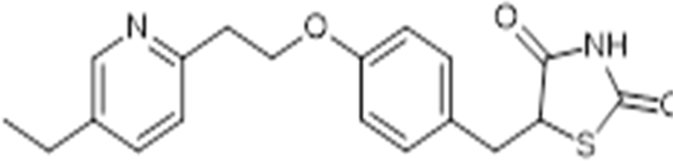

### 3.2 Root Mean Square Deviation analysis

**FIGURE 3:**
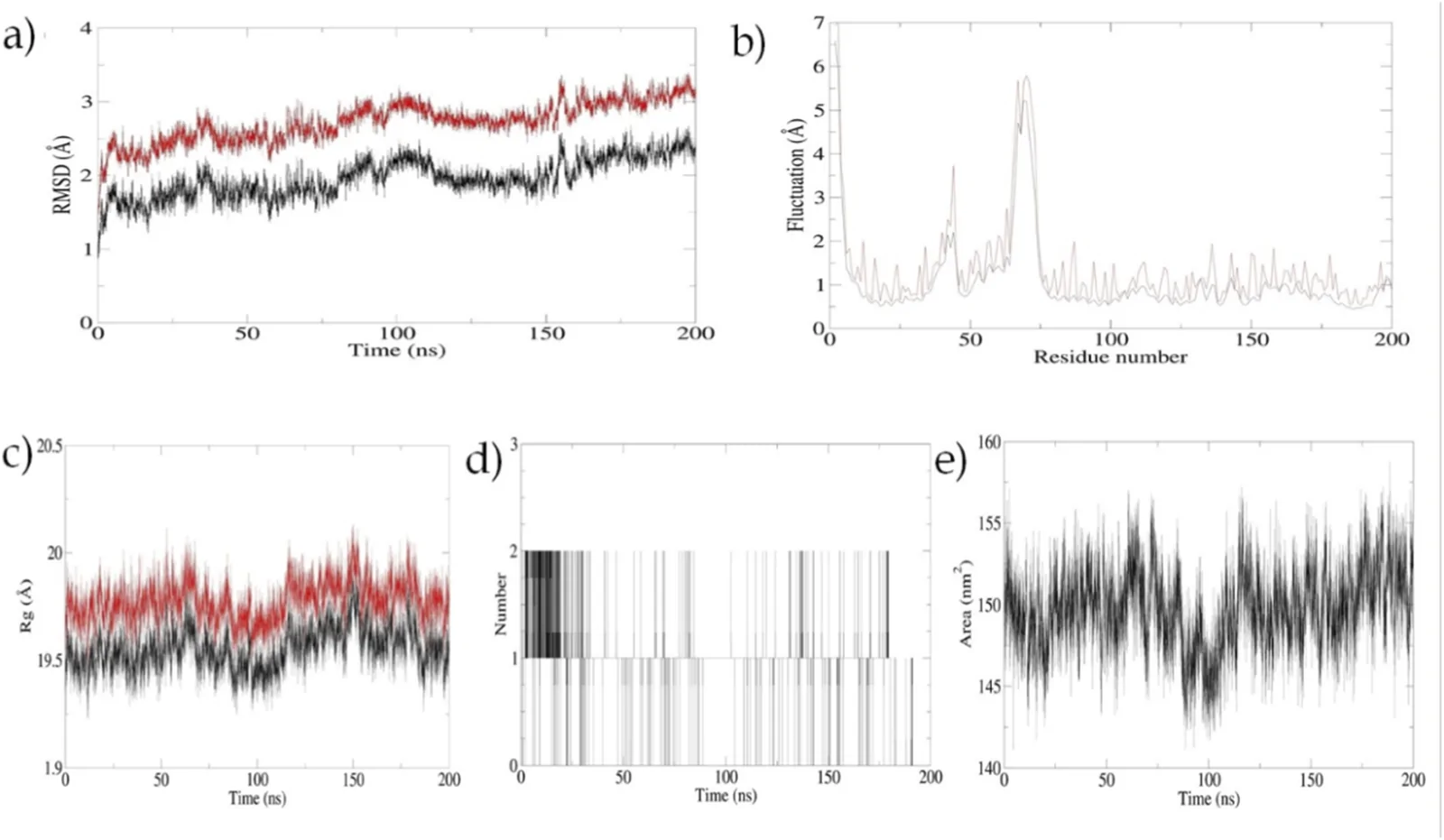
Illustrates the stability analysis of the C139-5 GTN complex: **(a)** RMSD of the complex, **(b)** RMSF of the Cα atoms (black) and the complex (red), **(c)** RoG for the protein backbone (black) and the complex (red), **(d)** Number of hydrogen bonds formed, **(e)** SASA for the protein (black) and the complex (red).

**FIGURE 4:**
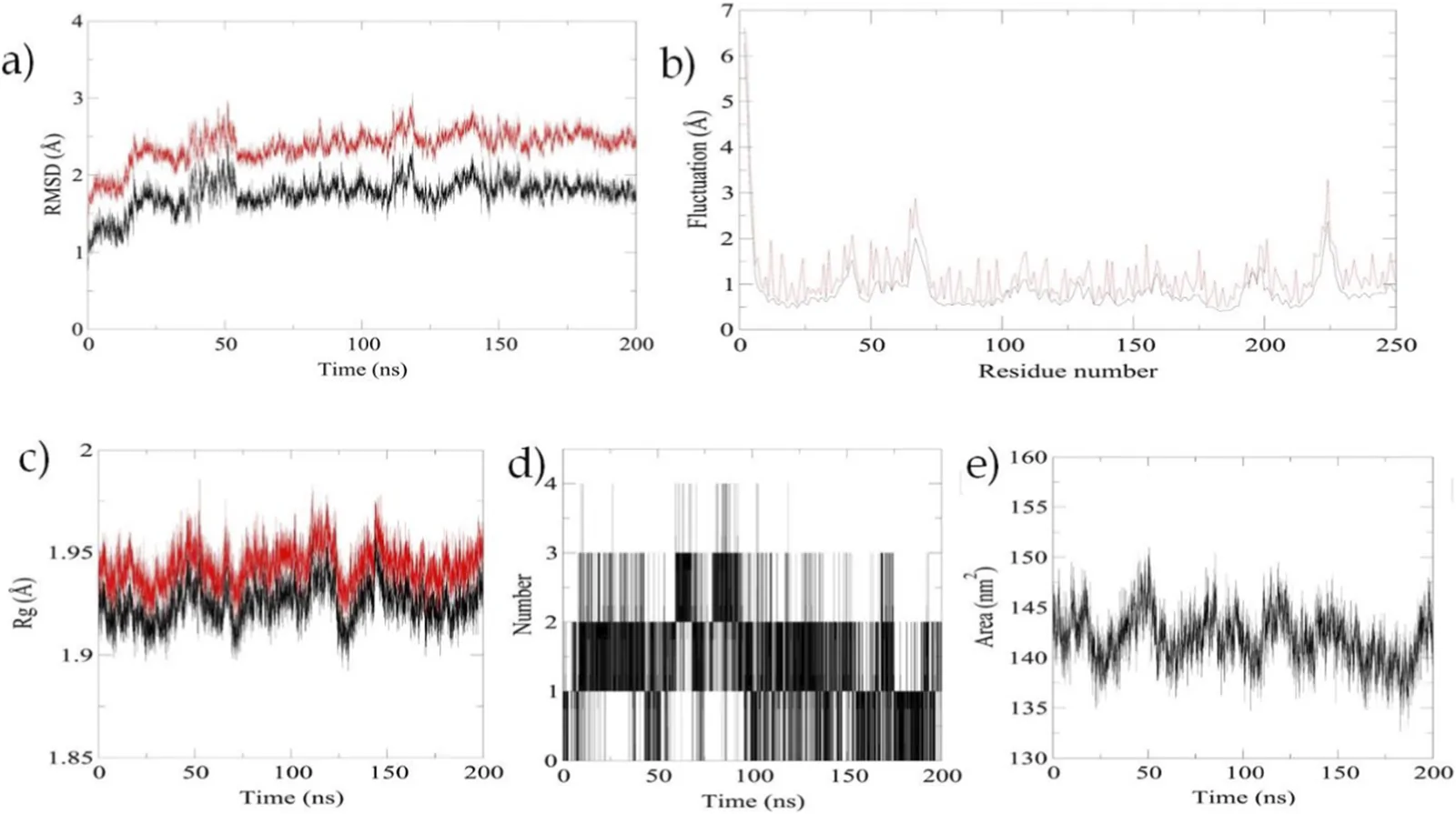
Illustrates the stability analysis of the Q-35-5 GT N complex: **(a)** RMSD of the complex, **(b)** RMSF of the Cα atoms (black) and the complex (red), **(c)** RoG for the protein backbone (black) and the complex (red), **(d)** Number of hydrogen bonds formed, **(e)** SASA for the protein (black) and the complex (red).

**FIGURE 5:**
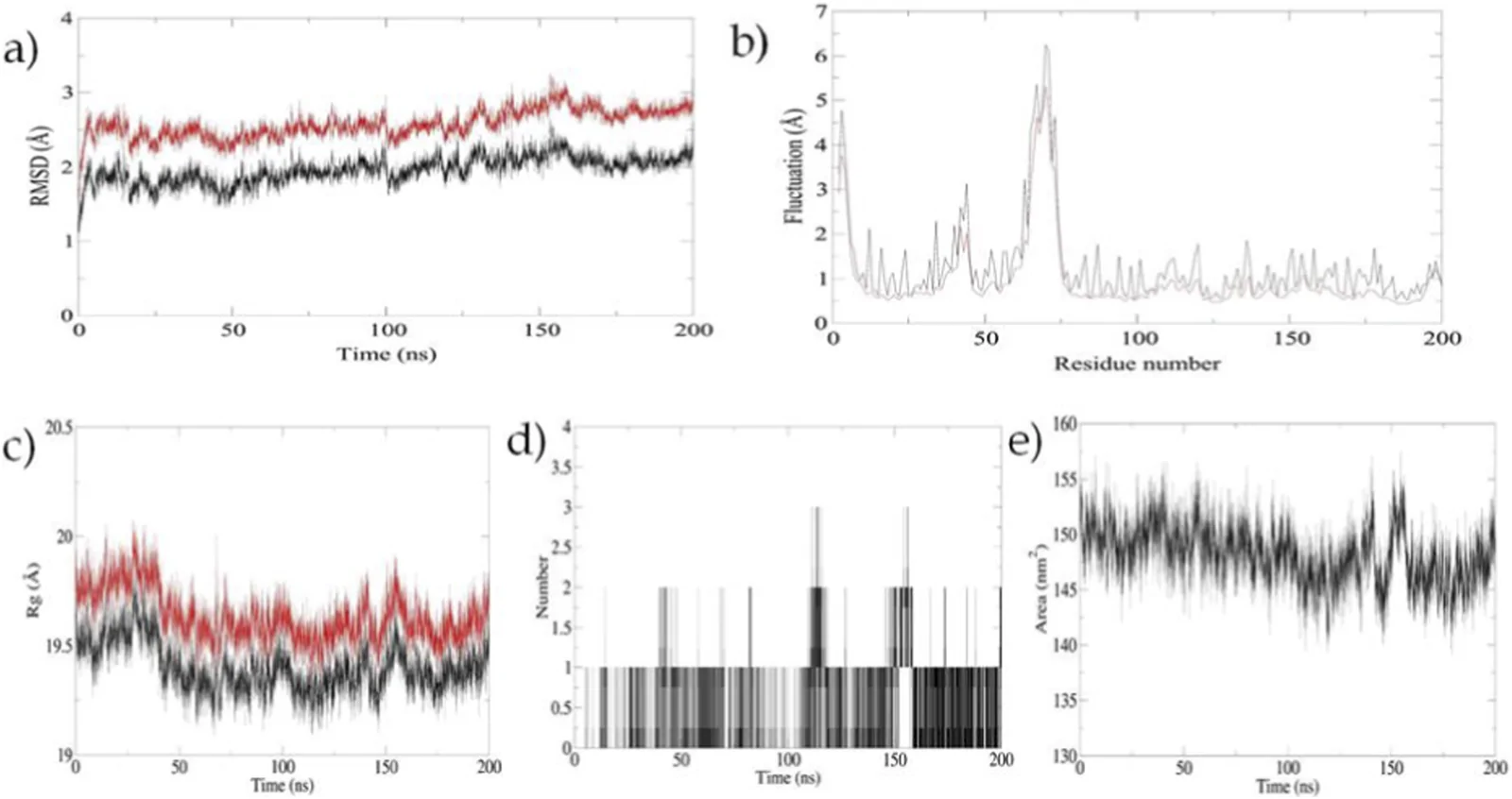
Illustrates the stability analysis of the (pioglitazone-5GTNcomplex: **(a)** RMSD of the complex, **(b)** RMSF of the Cα atoms (black) and the complex (red), **(c)** RoG for the protein backbone (black) and the complex (red), **(d)** Number of hydrogen bonds formed, **(e)** SASA for the protein (black) and the complex (red).

**FIGURE 6:**
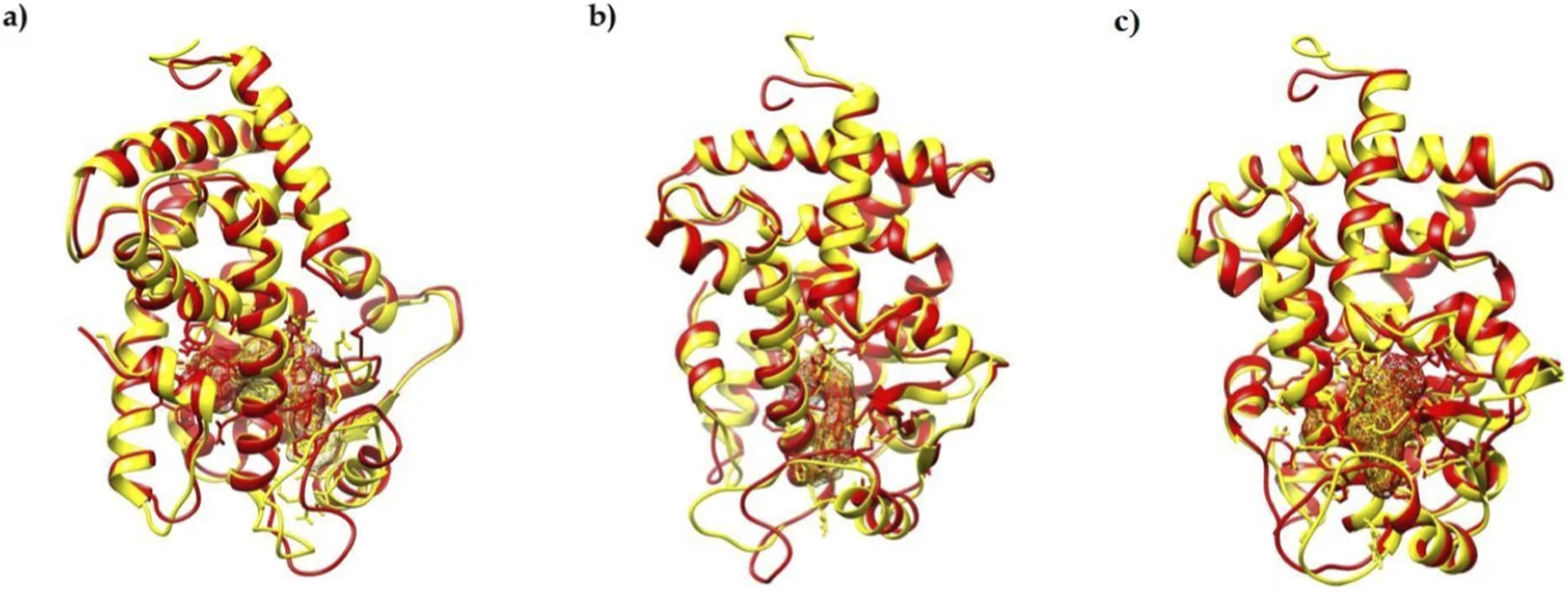
Illustrates the start (red) and end (yellow) interaction of the ligand with receptor **(a)** C139, **(b)** Pioglitazone, **(c)** Q-35 with 5 GTN.

The stability of the protein–ligand complexes was evaluated by analysing atomic displacements relative to their initial conformations over a 200 ns MD simulation. RMSD analysis was used to assess the structural integrity of the protein backbone and the persistence of ligand-binding interactions. The C139–5 GTN complex exhibited a maximum RMSD of 3.3 Å at 197 ns, with an average deviation of 2.3 Å. The Q-35–5 GT N complex showed a peak RMSD of 3.0 Å at 118 ns but maintained a notably lower average deviation of 0.6 Å, indicating rapid conformational convergence following initial fluctuations. In comparison, the pioglitazone–5GTN complex reached a maximum RMSD of 3.2 Å at 153 ns, with an average deviation of 2.4 Å. Overall, the Q-35–5 GT N complex exhibited the greatest stability among the three systems, as evidenced by its markedly lower average RMSD value. Although the C139–5 GTN complex displayed higher fluctuations than Q-35, its RMSD profile remained comparable to that of the pioglitazone–5GTN complex, suggesting sustained binding within the PPARγ ligand-binding pocket during the simulation. The RMSD profiles are illustrated in Figures 3a–5a, and the start and end interactions of the ligands C139, pioglitazone, and Q-35 with 5 GTN are illustrated in Figures 6a–c. A dynamic visualisation of the simulation is provided as supplementary material (MD movie).

### 3.3 Root Mean Square Fluctuation analysis

The residue-level flexibility of the protein–ligand complexes was evaluated using RMSF analysis over a 200 ns MD simulation. The C139–5 GTN complex exhibited RMSF values ranging from 0.5 Å to 7.0 Å, whereas the Q-35–5 GT N complex showed lower fluctuations, ranging from 1 Å to 3.5 Å. In contrast, the pioglitazone–5GTN complex exhibited a broader fluctuation profile, with RMSF values ranging from approximately 2.0–6.4 Å. The RMSF results indicate reduced residue mobility in the Q-35 complex, whereas C139 showed an intermediate fluctuation pattern compared with Q-35 and pioglitazone. The RMSF profiles are presented in (Figures 3b–5b).

### 3.4 Radius of Gyration analysis

This study assessed the compactness of protein–ligand complexes during MD simulations. The C139–5 GTN complex displayed RoG values ranging from 19.3 Å to 20.1 Å, whereas the Q-35–5 GT N complex ranged between 19.0 Å and 19.8 Å. In comparison, the pioglitazone–5GTN complex showed values ranging from 19.1 Å to 20.2 Å. The relatively narrow RoG distributions observed for all systems indicate minimal changes in overall protein compactness during the simulation. The RoG profiles are presented in Figures 3c–5c.

### 3.5 Hydrogen bond analysis

This study assessed the number and consistency of hydrogen bonds in receptor–ligand complexes throughout the 200 ns MD simulation. The 5 GTN–C139 complex consistently formed two hydrogen bonds, whereas the Q-35 complex maintained four hydrogen bonds throughout the simulation. The 5 GTN–pioglitazone complex exhibited a maximum of three hydrogen bonds, although these interactions fluctuated considerably. These results suggest stronger, more persistent intermolecular interactions for Q-35, whereas C139 maintained a moderate but consistent hydrogen-bonding pattern. The H-bond analysis is presented in Figures 3d–5d.

### 3.6 Solvent-accessible surface area analysis

The SASA study was performed to evaluate solvent exposure and ligand-induced conformational changes during the simulation. The SASA ranged from ^116.8 nm2^ to 141.3 nm^2^ for the C139–5 GTN complex, from 132 nm^2^ to 151 nm^2^ for the Q-35–5 GT N complex, and from 118 nm^2^ to 158 nm^2^ for the pioglitazone–5GTN complex. The observed SASA ranges suggest that no major changes in solvent exposure occurred during the simulation, indicating that the overall protein fold was preserved across all systems. The SASA analysis is presented in Figures 3e–5e.

### 3.7 Principal component analysis

**FIGURE 7:**
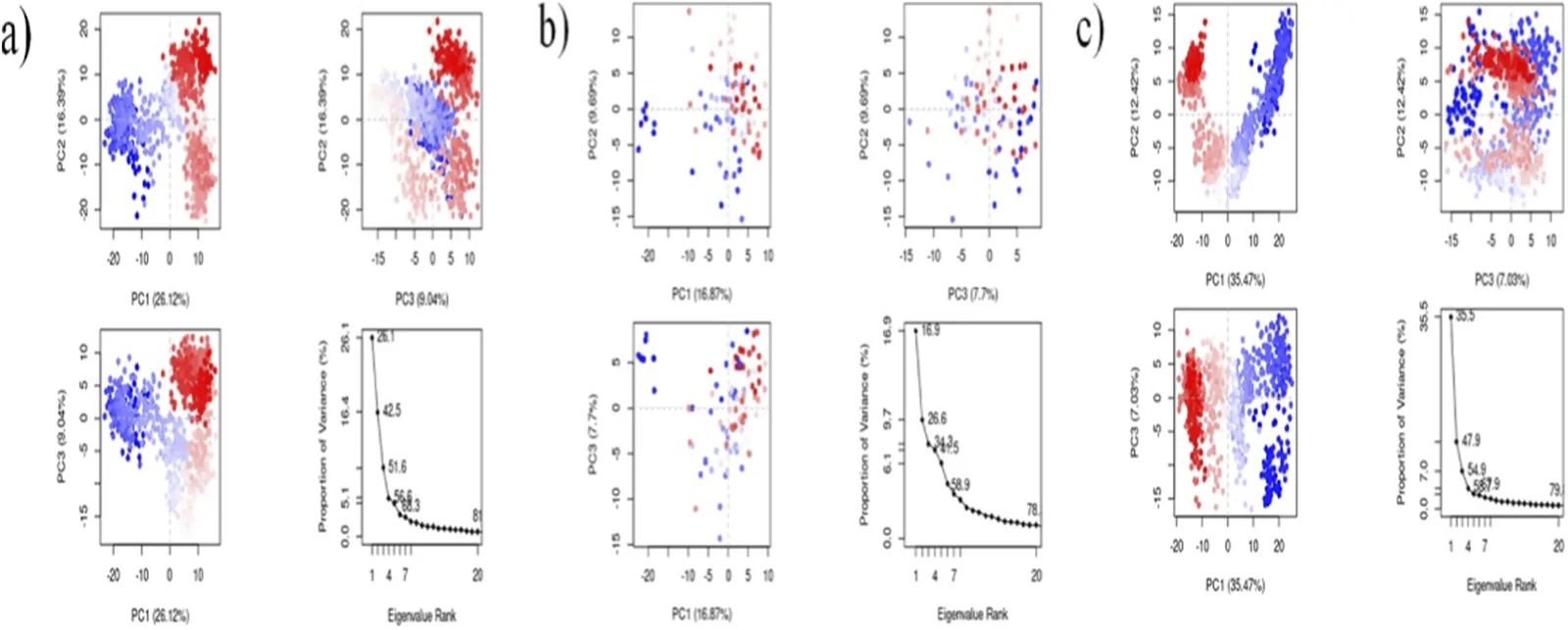
Principal component analysis for **(a)** C139-5 GTN **(b)** Q-35-5 GT N and **(c)** Pioglitazone-5 GTN.

The global conformational dynamics of the C139–5 GTN, Q-35–5 GT N, and pioglitazone–5GTN complexes were examined using PCA over the 200 ns simulation. The pioglitazone–5GTN complex showed the greatest contribution from the first principal component (PC1), accounting for 35.5% of the total variance, suggesting more extensive collective motions and broader conformational sampling. In contrast, the C139–5 GTN and Q-35–5 GT N complexes showed lower PC1 contributions of 26.1% and 16.9%, respectively. The conformational space projection revealed a broader distribution for pioglitazone, a confined cluster for Q-35, and an intermediate pattern for C139, consistent with the trends observed in RMSD and RMSF analyses. The PCA results for all three complexes are presented in Figure 7.

### 3.8 Dynamic cross-correlation map

**FIGURE 8:**
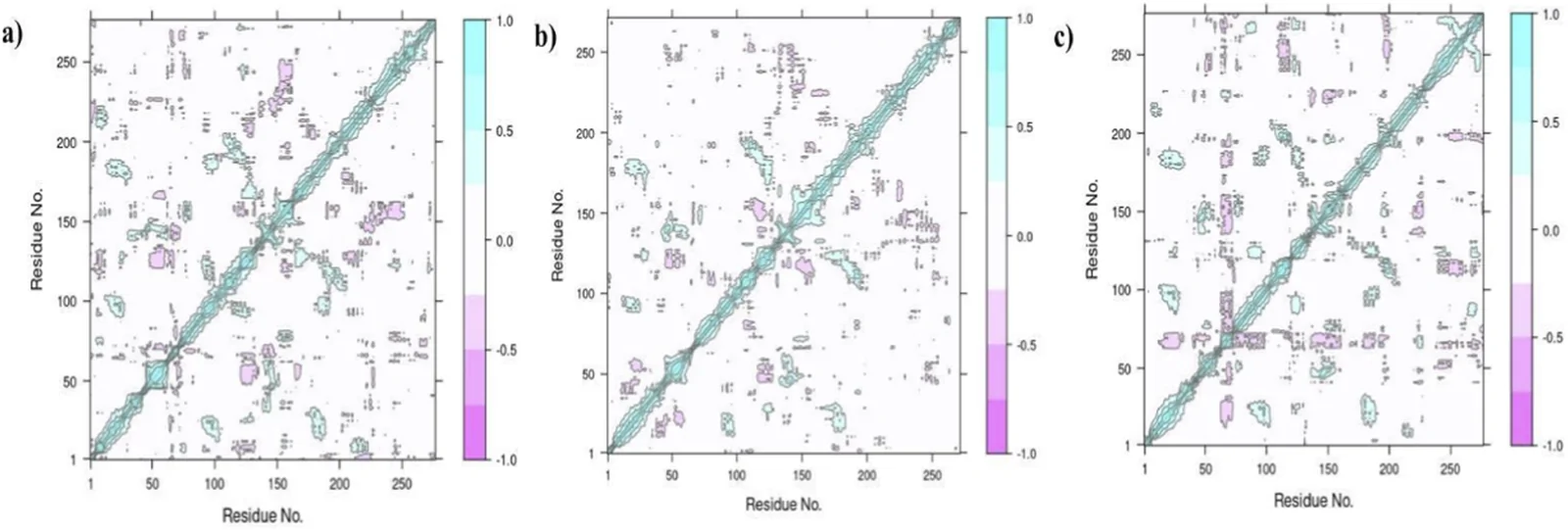
DCCM plots for amino acid residues present in **(a)** C139-5 GTN, **(b)** Q-35-5 GT N, and **(c)** Pioglitazone-5 GTN dynamic system.

The correlated motions of protein residues during the 200 ns MD simulation were evaluated using DCCM analysis. This approach quantifies the extent of coupled atomic fluctuations, with correlation coefficients ranging from −1 to +1. Positive values indicate concerted (parallel) motions between residue pairs, whereas negative values represent anti-correlated (opposing) movements. The C139–5 GTN complex exhibited pronounced positive correlations, particularly among residues spanning regions 60–140 and 180–280, suggesting coordinated motions within these segments. The Q-35–5 GT N complex showed more localised positive correlations, primarily involving residues 130–160 and 200–250, indicating region-specific dynamic coupling. In contrast, the pioglitazone–5GTN complex displayed a higher prevalence of anti-correlated motions across multiple regions, reflecting a distinct pattern of residue communication. Overall, the DCCM analysis revealed ligand-dependent differences in residue coupling and dynamic communication networks, complementing the conformational trends observed in the RMSD, RMSF, and PCA analyses. The corresponding DCCM profiles are presented in Figure 8.

### 3.9 Free-energy landscape

**FIGURE 9:**
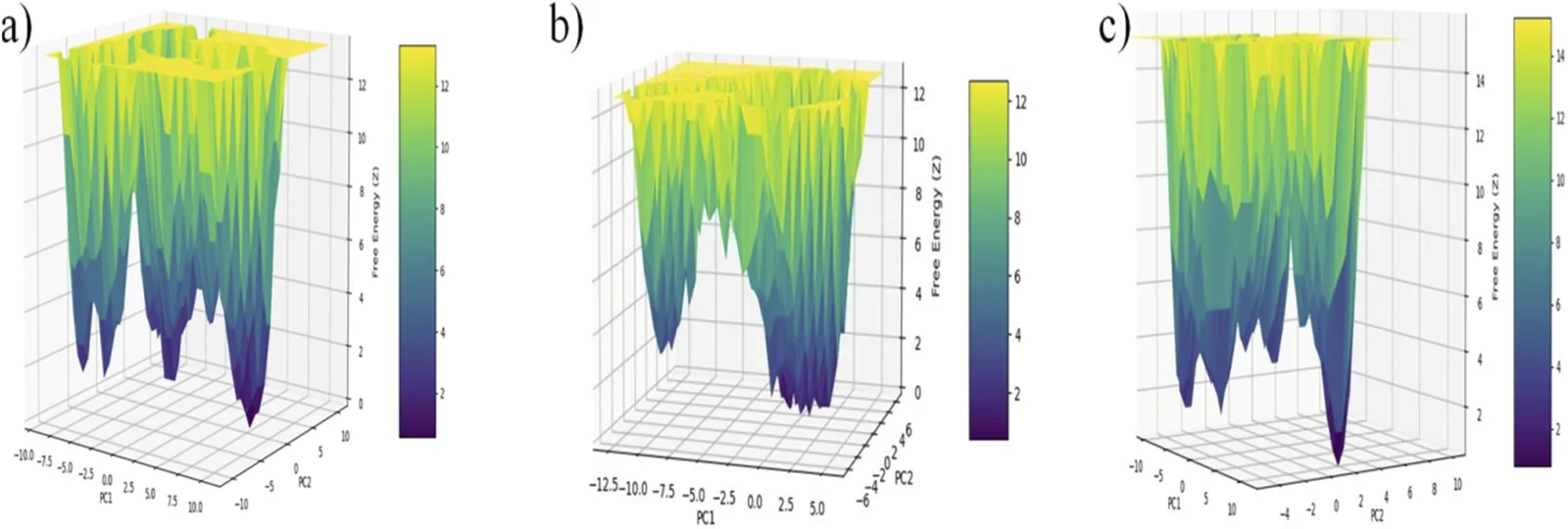
Graphical representation of the FEL for **(a)** C139-5 GTN **(b)** Q-35-5 GT N and **(c)** Pioglitazone-5 GTN.

This analysis was performed to characterise the conformational space sampled by the protein–ligand complexes during the MD simulations. The FEL provides a thermodynamic representation of the conformational ensemble by identifying energetically favorable states based on the first two components PC1 and PC2. The C139–5 GTN complex exhibited a Gibbs free energy range of 0–12 kJ/mol, whereas the pioglitazone–5GTN complex showed a broader range of 0–14 kJ/mol. Both the C139–5 GTN and Q-35–5 GT N complexes displayed multiple energy minima, indicating the sampling of several favorable conformational states during the simulation. The lowest free-energy basin for the C139–5 GTN complex was located at PC1 = −12.40 and PC2 = 1.21, while that of the Q-35–5 GT N complex was centered at PC1 = 2.06 and PC2 = 7.28. In contrast, the pioglitazone–5GTN complex exhibited its lowest free-energy basin at PC1 = 8.60 and PC2 = 0.64. These differences in energy-basin distribution reflect distinct conformational sampling patterns among the complexes and are consistent with the PCA results, which revealed more confined conformational space for Q-35 and intermediate conformational behavior for C139. The FEL graphical representation is shown in Figure 9. Collectively, the RMSD, RMSF, RoG, SASA, H-bond, PCA, DCCM and FEL analyses indicate that the Q-35–5 GT N complex exhibited the highest conformational stability, while the C139–5 GTN complex maintained stable binding behaviour comparable to the reference drug pioglitazone throughout the 200 ns simulation.

### 3.10 Molecular mechanics Poisson–Boltzmann surface area

**FIGURE 10:**
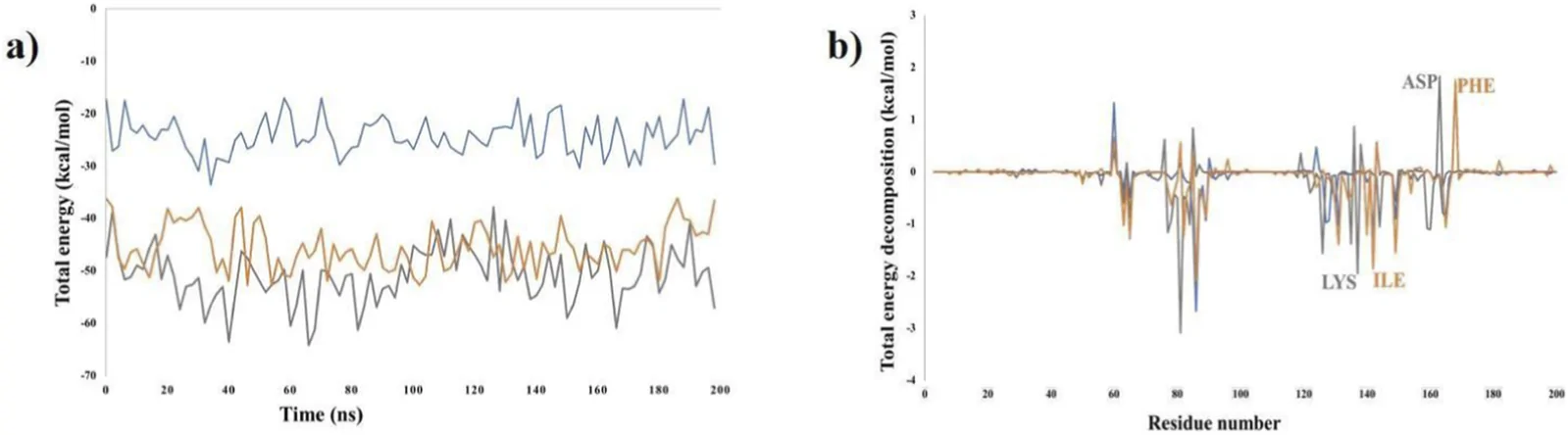
The energy decomposition of C139 (blue), pioglitazone (orange) and Q-35 (gray) with 5 GTN receptor where **(a)** total energy decomposition versus time and **(b)** total energy decomposition versus residue.

This analysis was performed to determine the total binding free energy and evaluate the relative stability of the C139–pioglitazone and Q-35 GTN complexes. Binding free energy calculations were conducted with over 1000 representative frames extracted from each MD trajectory. The MM/PBSA results confirmed that both ligands interact with the 5 GTN receptor through distinct molecular mechanical energy components. Per-residue energy-contribution analysis further identified key residues that bind the ligand in the C139–5 GTN complex. Among the three complexes, Q-35 exhibited a total binding free energy of −50.00 kcal/mol, Compound C139 also exhibited a strong binding affinity with a total binding free energy of −45.54 kcal/mol, and reference drug pioglitazone showed −24.31 kcal/mol. These computational results suggest that compounds Q-35 and C139 may possess more favourable predicted binding affinities toward the 5 GTN receptor than pioglitazone. However, the binding free energy estimated by the MM/PBSA method should be considered a predictive approximation, further validated experimentally. A detailed breakdown of the energy components and per-residue contributions is provided in Figure 10; Table 2.

**TABLE 2:** MMPBSA analysis of C139 with pioglitazone.

5 GTN complex	ΔVDWAALS	ΔEEL	ΔEPB	ΔENPOLAR	ΔGGAS	ΔGSOLV	ΔTOTAL
C139	−70.24	−8.81	39.63	−6.12	−79.04	33.51	−45.54
Q-35	−83.2	−23.62	63.5	−7.51	−106.82	55.99	−50.84
Pioglitazone	−51.09	−9.34	40.95	−4.83	−60.43	36.12	−24.31

ΔVDWAALS, van der Waals molecular mechanics energy; ΔEEL, energy of electrostatic molecular mechanics; ΔEPB, polar component of the energy of solvation; ΔENPOLAR: Non-polar portion of the solvation energy that comes from interactions between the solute and the solvent; ΔGGAS, total gas phase molecular mechanics energy; ΔGSOLV, total solvation energy; ΔGTotal: Total binding energy.

### 3.11 Physicochemical properties and predicted ADME profile

To evaluate the drug-like properties of selected compounds, physicochemical and pharmacokinetic properties were calculated using the QikProp module (Schrödinger, LLC). Various parameters were calculated to assess its bioavailability and safety, and all values were within permissible limits. The calculated metabolic reactions (number of metabolites, #metab) were in the range of two to four for all nine compounds. In contrast, both the Q-35 and pioglitazone exhibited #metab values of 6, suggesting a faster metabolic clearance of designed compounds. However, lower metab values reduce metabolic susceptibility with improved metabolic stability but also increase the risk of accumulation and associated toxicity. All selected compounds exhibited predicted human oral absorption of, indicating strong potential for efficient gastrointestinal uptake. In contrast, Q-35 demonstrated a significantly predicted absorption of 47.9%. Predicted binding affinity to human serum albumin (logKhsa) ranged from 0.448 to 1.266, with compound C139 exhibiting the highest value, suggesting strong plasma protein binding. Drug-likeness was further evaluated using the #star descriptor, which compares molecular properties to those of known drugs. All compounds exhibited scores ranging from 1 to 3, indicating within the acceptable range. The Caco-2 cell permeability values, which serve as a measure of intestinal absorption, ranged from 22.27 to 3441 nm/s, with compound C139 showing the highest permeability. The solvent-accessible surface area of hydrophobic regions (FOSA), computed using a 1.4 Å probe, remained within the recommended range for all compounds. Furthermore, according to Lipinski’s Rule of Five (Ro5), which assesses drug-likeness based on parameters such as logP (≤5), molecular weight (≤500 Da), hydrogen bond acceptors (≤10), and hydrogen bond donors (≤5), all compounds met the criteria except for C131 and C202, both showing one rule violation. However, Q-35 exhibited three Ro5 violations, indicating suboptimal oral drug-likeness. Overall, the findings identify compound C139 as the promising candidate, demonstrating optimal ADME characteristics and favourable pharmacokinetic properties. A comprehensive summary of all physicochemical and ADME parameters is provided in Table 3.

**TABLE 3:** Physicochemical properties and Predicted ADME profile.

Compound code	^a^stars	^b^metab	%HOA	QPPCaco	RoFV	QPlogKhsa	FOSA	Volume
C139	2	2	100	3441.62	1	1.266	195.90	1465.66
C184	2	2	100	2570.22	0	0.448	78.79	1302.57
C107	3	2	100	2568.74	2	0.565	115.87	1461.36
C131	3	2	100	2423.55	1	1.113	117.88	1441.24
C167	3	3	100	1379.82	1	0.669	79.41	1255.69
C113	1	4	100	937.49	0	0.714	78.83	1258.62
C202	3	2	100	2421.16	2	0.993	115.26	1491.34
C37	1	5	100	938.46	1	0.601	407.07	1297.08
C29	2	4	100	1376.47	0	0.555	177.73	1048.37
Q-35	3	6	47.9	22.27	3	1.00	464.47	1845.51
Pioglitazone (std)	0	6	100	655.62	0	0.493	257.93	1140.54

^a^ stars: Number of property that fall outside the 95% range of similar values for known drugs. (range: 0–5).

^b^ metab: Number of likely metabolic reactions (range: 1–8); %HOA:Percentage human oral absorption (range: >80% is high); QPPCaco, Projected apparent Caco-2, cell (range: 500 great); RoFV: Lipinski’s rule of five; QPlogKhsa: Estimate of binding to human serum albumin (range: −1.5–1.5); FOSA: SASA’s hydrophobic component (range: 0–750); Volume: solvent accessible in cubic angstroms by 1.4 Å radius probe (range: 500–2,000).

### 3.12 Generation of pharmacophore modeling

**FIGURE 11:**
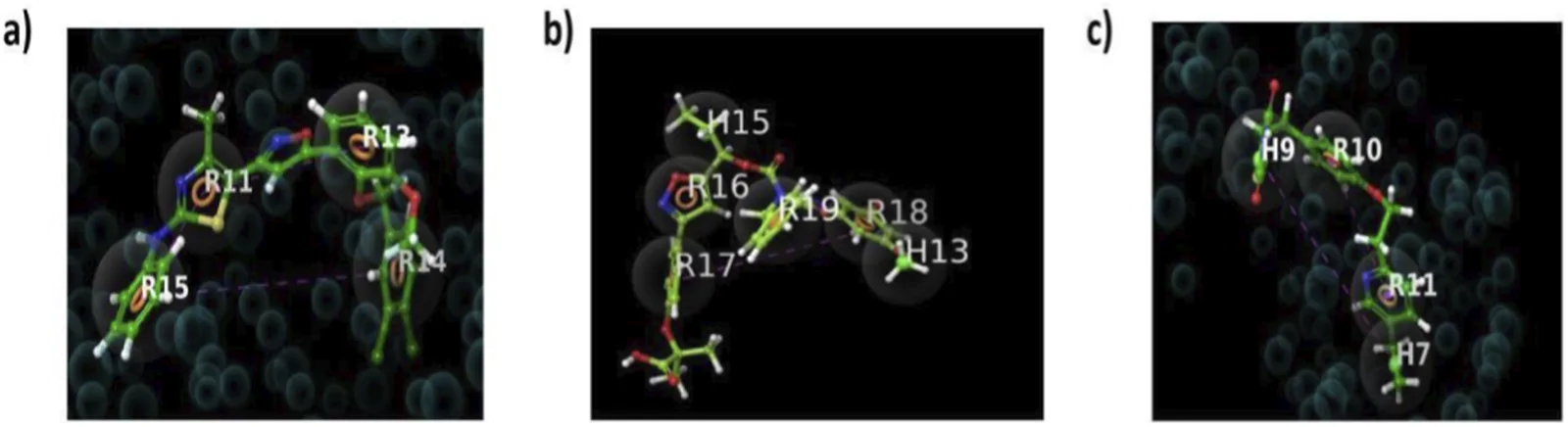
Pharmacophore model complex of **(a)** C139 **(b)** Q-35 and **(c)** Pioglitazone.

Pharmacophore modelling was performed to determine the essential steric, electronic and spatial arrangements of functional groups that contribute to the ligands’ biological activity within the receptor’s binding site. This approach facilitated the characterization of essential molecular interactions that govern ligand recognition and binding affinity. For compound C139, the pharmacophoric model revealed four aromatic ring features (R14, R13, R11, and R15), with interatomic distances measured as follows: R14–R13 (6.30 Å), R13–R11 (7.52 Å), R11–R15 (4.93 Å), and R15–R14 (11.70 Å). These spatial parameters underscore the presence of an extended aromatic system, which appears critical for stable and specific interactions within the PPARγ binding pocket. In contrast, the Q-35 displayed a pharmacophoric pattern comprising two hydrophobic features (H9 and H7) and four aromatic rings (R17, R16, R19, and R18), indicating a more complex interaction profile. Pioglitazone primarily engages the binding site through two hydrophobic moieties (H9 and H7) and two aromatic rings (R10 and R11). The pharmacophore models of compound C139, Q-35 and pioglitazone, as illustrated in Figure 11, clearly depict the differential arrangement and complexity of their interaction features, which are likely to contribute to their binding affinity and overall biological activity for compound C139.

### 3.13 PASS predicted value

PASS analysis was performed to estimate the potential antidiabetic activity of the top nine docked compounds. The predicted probability of activity (Pa) values was subsequently compared to that of the, pioglitazone and Q-35. The Pa values for the tested compounds ranged from 0.21 to 0.47. The Q-35 exhibited the highest Pa value of 0.63, indicating a strong likelihood of antidiabetic activity. Among the novel compounds, C139 and C29 demonstrated the highest predicted activities, with Pa values of 0.47 and 0.42, respectively. In comparison, the reference drug pioglitazone showed a lower Pa value of 0.41. Although compound C139 exhibited a moderate PASS-predicted activity (Pa = 0.47), which was lower than that of Q-35 but comparable to pioglitazone. These findings provide preliminary support for its antidiabetic activity and suggest that C139 may be considered a computationally prioritized lead candidate when evaluated in conjunction with its overall *in silico* profile. However, these findings require further experimental validation. The complete set of predicted Pa values for all compounds is presented in Table 4.

**TABLE 4:** Predicted anti-diabetic Pa (Pharmacologically active) value of top nine docked molecules.

Compound code	Pass value
C139	0.47
C184	0.25
C107	0.24
C131	0.36
C167	0.28
C113	0.31
C202	0.30
C37	0.21
C29	0.42
Q-35	0.63
Pioglitazone (std)	0.41

### 3.14 Toxicity prediction

Toxicity profiling of the top-ranked compounds was conducted using the ProTox 3.0 web server to predict acute oral toxicity and potential organ-specific toxicities. Co-crystal Q-35 exhibited a predicted LD_50_ value of 2500 mg/kg, while compound C139 showed a predicted LD_50_ of 3000 mg/kg. Based on the Globally Harmonized System (GHS) classification, both compounds were assigned to Toxicity Class V, indicating relatively low predicted acute toxicity. In comparison, the reference drug pioglitazone exhibited a predicted LD_50_ value of 1000 mg/kg and was classified as Toxicity Class IV. These results suggest lower predicted acute toxicity risks for C139 and Q-35 than for pioglitazone in the ProTox 3.0 model. Similarly, predictions of organ-specific toxicities, including neurotoxicity, clinical toxicity, and cardiotoxicity, were evaluated for all selected compounds. Among the evaluated compounds, C139 showed lower predicted probabilities of several organ-specific toxicities than pioglitazone. However, these findings are based on computational predictions and should be regarded as preliminary indicators of potential toxicological behaviour. Therefore, the predicted toxicity profiles require validation through appropriate *in vitro* and *in vivo* toxicological studies. A comprehensive summary of the toxicity predictions for all compounds is provided in Table 5.

**TABLE 5:** Toxicity prediction using ProTox 3.0 for the top nine docked molecules.

Comp code	LD50 (mg/kg)	Neurotoxicity	Probability	Clinical toxicity	Probability	Cardiotoxicity	Probability
C139	3000	I	0.57	I	0.51	I	0.68
C184	3000	A	0.57	A	0.50	I	0.85
C107	1000	I	0.54	I	0.53	I	0.67
C131	1000	I	0.65	I	0.51	I	0.67
C167	3000	I	0.58	A	0.52	I	0.68
C113	500	A	0.59	A	0.56	I	0.83
C202	1000	I	0.53	A	0.54	I	0.62
C37	1000	I	0.55	I	0.55	I	0.66
C29	800	A	0.56	I	0.52	I	0.84
Q-35	2500	A	0.87	I	0.56	I	0.77
Pioglitazone (std)	1000	A	0.83	A	0.86	I	0.63

## 4 Conclusion

In this study, thiazole-coupled isoxazole derivatives (C1–C210) were evaluated as potential PPARγ (5 GTN) modulators for antidiabetic therapy using integrated computational approaches and compared with Q-35 and pioglitazone. Among the designed compounds, C139 exhibited the most favourable binding affinity and demonstrated stable interactions throughout 200 ns MD simulations. MM-PBSA, PCA, DCCM, and FEL analyses further supported its stable binding behavior and favorable conformational dynamics. In addition, C139 showed acceptable predicted ADME properties, low toxicity, and higher predicted antidiabetic activity. Overall, C139 emerged as a computationally identified PPARγ modulator. However, these findings remain predictive and require experimental validation through *in vitro* and *in vivo* studies to confirm their biological activity and therapeutic potential. The study highlights the utility of *in silico* approaches for accelerating early-stage drug discovery.

## Data Availability

The original contributions presented in the study are included in the article/Supplementary Material; further inquiries can be directed to the corresponding author.

## Glossary

ADME: Absorption, Distribution, Metabolism, and Excretion, *CDK5* Cyclin-Dependent Kinase5
2D: 2 Dimensional
3D: 3 Dimensional
DCCM: Dynamic cross-correlation matrix
DOF: Degrees Of Freedom
DM: Diabetes mellitus
DPP-4: Dipeptidyl peptidase-4.
DSV: Discovery Studio Visualizer
FEL: Free Energy Landscape
GLP-1: Glucagon-Like Peptide-1
H-bond: Hydrogen Bond
**LD**_**50**_: Lethal Dose
MD: Molecular dynamics
MMPBSA: Molecular Mechanics Poisson–Boltzmann Surface Area
NVT: Number of particles, Volume, and Temperature ensemble
NPT: Number of particles, Pressure, and Temperature ensemble
PASS: Predicting Activity Spectra for Substances
PPARγ: Peroxisome Proliferator-Activated Receptor Gamma
H-bond: Hydrogen Bond
PCA: Principal Component Analysis
PC 1: First Principal Component
PB: Poisson–Boltzmann
PDB: Protein Data Bank
Pi: Pharmacological inactivity
Pa: Pharmacological activity
PME: Particle Mesh Ewald
RoG: Radius of gyration
Ro5: Rule of Five
RMSF: Root Mean Square Fluctuation
RMSD: Root Mean Square Deviation
RoG: Radius of gyration
Ro5: Rule of Five
SASA: Solvent-Accessible Surface Area
SGLT2: Sodium-Glucose Co-Transporter 2
SMILES: Simplified Molecular Input Line Entry System
T2DM: Type 2 Diabetes Mellitus
TIP3P: Transferable Intermolecular Potential with 3 Points
TZDs: Thiazolidinedione
XP: Extra Precision
