# Supplementary material for: Design and in silico evaluation of Thiazole–Isoxazole hybrids as PPARγ-Targeted antidiabetic agents
Source: Front Bioinform. 2026 Aug 11;6:1873262. doi: 10.3389/fbinf.2026.1873262 (PMC13503372; doi:10.3389/fbinf.2026.1873262)
Supplement: Supplementary file 1 [file Table1.docx]

**Supplementary file 1**

**Canonical smile and R1 and R2 of designed ligands**

| Sl no: | Comp code | Canonical smile of designed compound compounds | R1 | R2 |
| --- | --- | --- | --- | --- |
| 1 | C1 | CNC1=NC(C)=C(C2=NOC(C3=CC=CC=C3)=C2)S1 | H | H |
| 2 | C2 | CNC1=NC(C)=C(C2=NOC(C3=C(O)C=CC=C3)=C2)S1 | H | 2-OH |
| 3 | C3 | CNC1=NC(C)=C(C2=NOC(C3=C(Cl)C=CC=C3)=C2)S1 | H | 2-Cl |
| 4 | C4 | CNC1=NC(C)=C(C2=NOC(C3=CC(Cl)=CC=C3)=C2)S1 | H | 3-Cl |
| 5 | C5 | CNC1=NC(C)=C(C2=NOC(C3=CC=C(Cl)C=C3)=C2)S1 | H | 4-Cl |
| 6 | C6 | CNC1=NC(C)=C(C2=NOC(C3=C(Cl)C=C(Cl)C=C3)=C2)S1 | H | 2,4-Cl |
| 7 | C7 | CNC1=NC=C(C2=NOC(C3=C(Cl)C=CC=C3Cl)=C2)S1 | H | 2,6-Cl |
| 8 | C8 | CNC1=NC=C(C2=NOC(C3=C(Cl)C(Cl)=CC=C3)=C2)S1 | H | 2,3-Cl |
| 9 | C9 | CNC1=NC=C(C2=NOC(C3=CC(Cl)=CC(Cl)=C3)=C2)S1 | H | 3,5-Cl |
| 10 | C10 | CNC1=NC=C(C2=NOC(C3=CC(Cl)=C(Cl)C=C3)=C2)S1 | H | 3,4-Cl |
| 11 | C11 | CNC1=NC=C(C2=NOC(C3=CC([N+]([O-])=O)=CC=C3)=C2)S1 | H | 3-NO_2_ |
| 12 | C12 | CNC1=NC=C(C2=NOC(C3=CC=C([N+]([O-])=O)C=C3)=C2)S1 | H | 4-NO_2_ |
| 13 | C13 | CNC1=NC=C(C2=NOC(C3=C([N+]([O-])=O)C=CC=C3)=C2)S1 | H | 2-NO_2_ |
| 14 | C14 | CNC1=NC=C(C2=NOC(C3=C([N+]([O-])=O)C=C([N+]([O-])=O)C=C3)=C2)S1 | H | 2,4-NO_2_ |
| 15 | C15 | CNC1=NC=C(C2=NOC(C3=C([N+]([O-])=O)C=CC=C3[N+]([O-])=O)=C2)S1 | H | 2,6-NO_2_ |
| 16 | C16 | CNC1=NC=C(C2=NOC(C3=CC([N+]([O-])=O)=CC([N+]([O-])=O)=C3)=C2)S1 | H | 3,5-NO_2_ |
| 17 | C17 | CNC1=NC=C(C2=NOC(C3=CC(C)=C(C)C(C)=C3)=C2)S1 | H | 3,4,5-CH_3_ |
| 18 | C18 | CNC1=NC=C(C2=NOC(C3=C(OC)C=CC=C3)=C2)S1 | H | 2-OCH_3_ |
| 19 | C19 | CNC1=NC=C(C2=NOC(C3=CC=C(OC)C=C3)=C2)S1 | H | 4-OCH_3_ |
| 20 | C20 | CNC1=NC=C(C2=NOC(C3=C(OC)C=CC(OC)=C3)=C2)S1 | H | 2,5-OCH_3_ |
| 21 | C21 | CNC1=NC=C(C2=NOC(C3=CC(OC)=C(OC)C=C3)=C2)S1 | H | 3,4-OCH_3_ |
| 22 | C22 | CNC1=NC=C(C2=NOC(C3=CC=C(N(C)C)C=C3)=C2)S1 | H | 4-N(CH_3_)_2_ |
| 23 | C23 | CNC1=NC=C(C2=NOC(C3=CC=C(F)C=C3)=C2)S1 | H | 4-F |
| 24 | C24 | CNC1=NC=C(C2=NOC(C3=CC=C(Br)C=C3)=C2)S1 | H | 4-Br |
| 25 | C25 | CNC1=NC(C)=C(C2=NOC(C3=CC=C(O)C=C3)=C2)S1 | H | 4-OH |
| 26 | C26 | CNC1=NC=C(C2=NOC(C3=CC(OC)=C(OC)C(OC)=C3)=C2)S1 | H | 3,4,5-OCH_3_ |
| 27 | C27 | CNC1=NC(C)=C(C2=NOC(C3=CC(OC)=C(O)C=C3)=C2)S1 | H | 3-OCH_3_,4-Cl |
| 28 | C28 | CNC1=NC(C)=C(C2=NOC(C3=C(Cl)C=CC(C(F)(F)F)=C3)=C2)S1 | H | 2-Cl, 5-CF_3_ |
| 29 | C29 | CNC1=NC(C)=C(C2=NOC(C3=CC=C(C(F)(F)F)C=C3)=C2)S1 | H | 4-CF_3_ |
| 30 | C30 | CNC1=NC(C)=C(C2=NOC(C3=C(C(F)(F)F)C=CC=C3)=C2)S1 | H | 2-CF_3_ |
| 31 | C31 | CNC1=NC(C)=C(C2=NOC(C3=C(Cl)C=C(N)C=C3)=C2)S1 | H | 2-Cl,4-NH_3_ |
| 32 | C32 | CNC1=NC(C)=C(C2=NOC(C3=CC(OC)=C(O)C([N+]([O-])=O)=C3)=C2)S1 | H | 3-NO_2_,4-OH,5- OCH_3_ |
| 33 | C33 | CNC1=NC=C(C2=NOC(C3=C(Br)C=C(O)C(OC)=C3)=C2)S1 | H | 2-Br,4-OH,5- OCH_3_ |
| 34 | C34 | CNC1=NC(C)=C(C2=NOC(C3=CC(OC)=C(O)C(Br)=C3)=C2)S1 | H | 3-Br,4-OH,5- OCH_3_ |
| 35 | C35 | CNC1=NC=C(C2=NOC(C3=CC(Br)=C(O)C(OCC)=C3)=C2)S1 | H | 3-Br,4-OH 5-C_2_H_5_O |
| 36 | C36 | CNC1=NC(C)=C(C2=NOC(C3=CC(OC)=C(O)C(I)=C3)=C2)S1 | H | 3- OCH_3_,4-OH,5-I |
| 37 | C37 | CNC1=NC=C(C2=NOC(C3=CC(Br)=C(OCCCCC)C(OC)=C3)=C2)S1 | H | 3-Br,4-C_5_H_12_O,5-OCH_3_ |
| 38 | C38 | CNC1=NC=C(C2=NOC(C3=C(Br)C(OCC4=CC=CC=C4)=C(OC)C=C3)=C2)S1 | H | 2-Br,3-C_7_H_7_O,4-OCH_3_ |
| 39 | C39 | CNC1=NC(C)=C(C2=NOC(C3=CC=C(OCC4=CC=CC=C4)C=C3)=C2)S1 | H | 4- C_7_H_7_O |
| 40 | C40 | CNC1=NC=C(C2=NOC(C3=CC(F)=C(OC(F)(F)F)C=C3)=C2)S1 | H | 3-F,4-OCF_3_ |
| 41 | C41 | CNC1=NC(C)=C(C2=NOC(C3=C(F)C=C(N4CCCOC4)C=C3)=C2)S1 | H | 2-F,4-C_4_H_8_NO |
| 42 | C42 | CNC1=NC=C(C2=NOC(C3=CC(F)=C(S(C)(=O)=O)C=C3)=C2)S1 | H | 3-F, 4-CH_3_O_2_S |
| 43 | C43 | CNC1=NC=C(C2=NOC(C3=CC(F)=CC=C3N4N=CC=C4)=C2)S1 | H | 2-C_3_H_3_N_2_,5-F |
| 44 | C44 | CNC1=NC(C)=C(C2=NOC(C3=CC=CC=C3C4=CN=CN=C4)=C2)S1 | H | 2-C_4_H_4_N_2_ |
| 45 | C45 | CNC1=NC(C)=C(C2=NOC(C3=C(C)C=C(N4CCCC4)C(F)=C3)=C2)S1 | H | 2-CH_3_ ,4-C_4_H_8_N,5-F |
| 46 | C46 | CNC1=NC=C(C2=NOC(C3=CC(OC)=CC=C3)=C2)S1 | H | 3-OCH_3_ |
| 47 | C47 | CNC1=NC(C)=C(C2=NOC(C3=C(O)C=CC([N+]([O-])=O)=C3)=C2)S1 | H | 2-OH, 5-NO_2_ |
| 48 | C48 | CNC1=NC(C)=C(C2=NOC(C3=C([N+]([O-])=O)C=CC(O)=C3)=C2)S1 | H | 2- NO_2_, 5-OH |
| 49 | C49 | CNC1=NC(C)=C(C2=NOC(C3=C([N+]([O-])=O)C=CC(Cl)=C3)=C2)S1 | H | 2- NO_2_,5-Cl |
| 50 | C50 | CNC1=NC(C)=C(C2=NOC(C3=C(OC)C=C([N+]([O-])=O)C=C3)=C2)S1 | H | 2-OCH_3_,4-NO_2_ |
| 51 | C51 | CNC1=NC(C)=C(C2=NOC(C3=C([N+]([O-])=O)C(OC)=CC=C3)=C2)S1 | H | 2-NO_2_ ,3-OCH_3_, |
| 52 | C52 | CNC1=NC=C(C2=NOC(C3=C(Cl)C=CC([N+]([O-])=O)=C3)=C2)S1 | H | 2-Cl, 5-NO_2_ |
| 53 | C53 | CNC1=NC=C(C2=NOC(C3=C([N+]([O-])=O)C=CC(OC)=C3)=C2)S1 | H | 2- NO_2,_ 5-OCH_3_ |
| 54 | C54 | CNC1=NC=C(C2=NOC(C3=C(O)C([N+]([O-])=O)=CC=C3)=C2)S1 | H | 2-OH, 3-NO_2_ |
| 55 | C55 | CNC1=NC=C(C2=NOC(C3=C(O)C([N+]([O-])=O)=CC(OC)=C3)=C2)S1 | H | 2-OH,3-NO_2_,5-OCH_3_ |
| 56 | C56 | CNC1=NC=C(C2=NOC(C3=C([N+]([O-])=O)C(OC)=CC=C3[N+]([O-])=O)=C2)S1 | H | 2,6-NO_2_, 3-OCH_3_ |
| 57 | C57 | CNC1=NC=C(C2=NOC(C3=C(Cl)C(Cl)=CC(Cl)=C3)=C2)S1 | H | 2,3,5-Cl |
| 58 | C58 | CNC1=NC(C)=C(C2=NOC(C3=CC(OC)=C(F)C=C3)=C2)S1 | H | 3-OCH_3_, 4-F |
| 59 | C59 | IC1=C(OC)C=CC(C2=CC(C3=CN=C(NC)S3)=NO2)=C1 | H | 3-I, 4-OCH_3_ |
| 60 | C60 | CNC1=NC=C(C2=NOC(C3=C(Cl)C(OC)=CC=C3Cl)=C2)S1 | H | 2,6-Cl, 3-OCH_3_ |
| 61 | C61 | CNC1=NC=C(C2=NOC(C3=CC(OC(C)C)=C(OC)C=C3)=C2)S1 | H | 3-C_3_H_7_O, 4-OCH_3_ |
| 62 | C62 | CNC1=NC=C(C2=NOC(C3=C(OCC4=CC=CC(Cl)=C4)C(OC)=CC=C3)=C2)S1 | H | 2-C_7_H_6_ClO, 3-OCH_3_ |
| 63 | C63 | CNC1=NC=C(C2=NOC(C3=CC(OCC)=C(OC)C=C3)=C2)S1 | H | 3-OC_2_H_5_ , 4-OCH_3_ |
| 64 | C64 | CNC1=NC(C)=C(C2=NOC(C3=CC(F)=C(O)C(OC)=C3)=C2)S1 | H | 3-F, 4-OH, 5-OCH_3_ |
| 65 | C65 | CNC1=NC(C)=C(C2=NOC(C3=CC(F)=C(OC)C=C3)=C2)S1 | H | 3-F, 4- OCH_3_ |
| 66 | C66 | CNC1=NC(C)=C(C2=NOC(C3=CC(Cl)=C(OC)C=C3)=C2)S1 | H | 3-Cl, 4- OCH_3_ |
| 67 | C67 | CNC1=NC(C)=C(C2=NOC(C3=C(Br)C(O)=C(OC)C=C3)=C2)S1 | H | 2-Br, 3-OH, 4- OCH_3_ |
| 68 | C68 | CNC1=NC(C)=C(C2=NOC(C3=CC(OC)=C(OCC4=CC=CC=C4)C=C3)=C2)S1 | H | 3- OCH_3_ ,4-C_7_H_7_O |
| 69 | C69 | CNC1=NC(C)=C(C2=NOC(C3=CC(Br)=C(OC(C)C)C(OC)=C3)=C2)S1 | H | 3-Br, 4-C_3_H_7_O, 5-OCH_3_ |
| 70 | C70 | CNC1=NC(C)=C(C2=NOC(C3=C(OCC4=CC(Cl)=C(Cl)C=C4)C(OC)=CC=C3)=C2)S1 | H | 2-C_7_H_5_Cl_2_O, 3-OCH_3_ |
| 71 | C71 | CC1=C(C2=NOC(C3=CC=CC=C3)=C2)SC(NC4=CC=CC=C4)=N1 | C_6_H_6_ | H |
| 72 | C72 | CC1=C(C2=NOC(C3=C(O)C=CC=C3)=C2)SC(NC4=CC=CC=C4)=N1 | C_6_H_6_ | 2-OH |
| 73 | C73 | CC1=C(C2=NOC(C3=C(Cl)C=CC=C3)=C2)SC(NC4=CC=CC=C4)=N1 | C_6_H_6_ | 2-Cl |
| 74 | C74 | CC1=C(C2=NOC(C3=CC(Cl)=CC=C3)=C2)SC(NC4=CC=CC=C4)=N1 | C_6_H_6_ | 3-Cl |
| 75 | C75 | CC1=C(C2=NOC(C3=CC=C(Cl)C=C3)=C2)SC(NC4=CC=CC=C4)=N1 | C_6_H_6_ | 4-Cl |
| 76 | C76 | CC1=C(C2=NOC(C3=C(Cl)C=C(Cl)C=C3)=C2)SC(NC4=CC=CC=C4)=N1 | C_6_H_6_ | 2,4-Cl |
| 77 | C77 | ClC1=CC=CC(Cl)=C1C2=CC(C3=CN=C(NC4=CC=CC=C4)S3)=NO2 | C_6_H_6_ | 2,6-Cl |
| 78 | C78 | ClC(C(Cl)=CC=C1)=C1C2=CC(C3=CN=C(NC4=CC=CC=C4)S3)=NO2 | C_6_H_6_ | 2,3-Cl |
| 79 | C79 | ClC1=CC(Cl)=CC(C2=CC(C3=CN=C(NC4=CC=CC=C4)S3)=NO2)=C1 | C_6_H_6_ | 3,5-Cl |
| 80 | C80 | ClC1=C(Cl)C=CC(C2=CC(C3=CN=C(NC4=CC=CC=C4)S3)=NO2)=C1 | C_6_H_6_ | 3,4-Cl |
| 81 | C81 | O=[N+](C1=CC=CC(C2=CC(C3=CN=C(NC4=CC=CC=C4)S3)=NO2)=C1)[O-] | C_6_H_6_ | 3-NO_2_ |
| 82 | C82 | O=[N+](C(C=C1)=CC=C1C2=CC(C3=CN=C(NC4=CC=CC=C4)S3)=NO2)[O-] | C_6_H_6_ | 4-NO_2_ |
| 83 | C83 | O=[N+](C(C=CC=C1)=C1C2=CC(C3=CN=C(NC4=CC=CC=C4)S3)=NO2)[O-] | C_6_H_6_ | 2-NO_2_ |
| 84 | C84 | O=[N+](C(C=C([N+]([O-])=O)C=C1)=C1C2=CC(C3=CN=C(NC4=CC=CC=C4)S3)=NO2)[O-] | C_6_H_6_ | 2,4-NO_2_ |
| 85 | C85 | O=[N+](C(C=CC=C1[N+]([O-])=O)=C1C2=CC(C3=CN=C(NC4=CC=CC=C4)S3)=NO2)[O-] | C_6_H_6_ | 2,6-NO_2_ |
| 86 | C86 | O=[N+](C1=CC([N+]([O-])=O)=CC(C2=CC(C3=CN=C(NC4=CC=CC=C4)S3)=NO2)=C1)[O-] | C_6_H_6_ | 3,5-NO_2_ |
| 87 | C87 | CC1=C(C)C(C)=CC(C2=CC(C3=CN=C(NC4=CC=CC=C4)S3)=NO2)=C1 | C_6_H_6_ | 3,4,5-CH_3_ |
| 88 | C88 | COC(C=CC=C1)=C1C2=CC(C3=CN=C(NC4=CC=CC=C4)S3)=NO2 | C_6_H_6_ | 2-OCH_3_ |
| 89 | C89 | COC(C=C1)=CC=C1C2=CC(C3=CN=C(NC4=CC=CC=C4)S3)=NO2 | C_6_H_6_ | 4-OCH_3_ |
| 90 | C90 | COC(C=CC(OC)=C1)=C1C2=CC(C3=CN=C(NC4=CC=CC=C4)S3)=NO2 | C_6_H_6_ | 2,5-OCH_3_ |
| 91 | C91 | COC1=C(OC)C=CC(C2=CC(C3=CN=C(NC4=CC=CC=C4)S3)=NO2)=C1 | C_6_H_6_ | 3,4-OCH_3_ |
| 92 | C92 | CN(C)C(C=C1)=CC=C1C2=CC(C3=CN=C(NC4=CC=CC=C4)S3)=NO2 | C_6_H_6_ | 4-N(CH_3_)_2_ |
| 93 | C93 | FC(C=C1)=CC=C1C2=CC(C3=CN=C(NC4=CC=CC=C4)S3)=NO2 | C_6_H_6_ | 4-F |
| 94 | C94 | BrC(C=C1)=CC=C1C2=CC(C3=CN=C(NC4=CC=CC=C4)S3)=NO2 | C_6_H_6_ | 4-Br |
| 95 | C95 | CC1=C(C2=NOC(C3=CC=C(O)C=C3)=C2)SC(NC4=CC=CC=C4)=N1 | C_6_H_6_ | 4-OH |
| 96 | C96 | COC1=C(OC)C(OC)=CC(C2=CC(C3=CN=C(NC4=CC=CC=C4)S3)=NO2)=C1 | C_6_H_6_ | 3,4,5-OCH_3_ |
| 97 | C97 | CC1=C(C2=NOC(C3=CC(OC)=C(O)C=C3)=C2)SC(NC4=CC=CC=C4)=N1 | C_6_H_6_ | 3-OCH_3_,4-Cl |
| 98 | C98 | CC1=C(C2=NOC(C3=C(Cl)C=CC(C(F)(F)F)=C3)=C2)SC(NC4=CC=CC=C4)=N1 | C_6_H_6_ | 2-Cl, 5-CF_3_ |
| 99 | C99 | CC1=C(C2=NOC(C3=CC=C(C(F)(F)F)C=C3)=C2)SC(NC4=CC=CC=C4)=N1 | C_6_H_6_ | 4-CF_3_ |
| 100 | C100 | CC1=C(C2=NOC(C3=C(C(F)(F)F)C=CC=C3)=C2)SC(NC4=CC=CC=C4)=N1 | C_6_H_6_ | 2-CF_3_ |
| 101 | C101 | CC1=C(C2=NOC(C3=C(Cl)C=C(N)C=C3)=C2)SC(NC4=CC=CC=C4)=N1 | C_6_H_6_ | 2-Cl,4-NH_3_ |
| 102 | C102 | CC1=C(C2=NOC(C3=CC(OC)=C(O)C([N+]([O-])=O)=C3)=C2)SC(NC4=CC=CC=C4)=N1 | C_6_H_6_ | 3-NO_2_,4-OH,5- OCH_3_ |
| 103 | C103 | BrC(C=C(O)C(OC)=C1)=C1C2=CC(C3=CN=C(NC4=CC=CC=C4)S3)=NO2 | C_6_H_6_ | 2-Br,4-OH,5- OCH_3_ |
| 104 | C104 | CC1=C(C2=NOC(C3=CC(OC)=C(O)C(Br)=C3)=C2)SC(NC4=CC=CC=C4)=N1 | C_6_H_6_ | 3-Br,4-OH,5- OCH_3_ |
| 105 | C105 | OC(C(OCC)=C1)=C(Br)C=C1C2=CC(C3=CN=C(NC4=CC=CC=C4)S3)=NO2 | C_6_H_6_ | 3-Br,4-OH 5-C_2_H_5_O |
| 106 | C106 | CC1=C(C2=NOC(C3=CC(OC)=C(O)C(I)=C3)=C2)SC(NC4=CC=CC=C4)=N1 | C_6_H_6_ | 3- OCH_3_,4-OH,5-I |
| 107 | C107 | BrC1=C(OCCCCC)C(OC)=CC(C2=CC(C3=CN=C(NC4=CC=CC=C4)S3)=NO2)=C1 | C_6_H_6_ | 3-Br,4-C_5_H_12_O,5-OCH_3_ |
| 108 | C108 | COC(C=C1)=C(OCC2=CC=CC=C2)C(Br)=C1C3=CC(C4=CN=C(NC5=CC=CC=C5)S4)=NO3 | C_6_H_6_ | 2-Br,3-C_7_H_7_O,4-OCH_3_ |
| 109 | C109 | CC1=C(C2=NOC(C3=CC=C(OCC4=CC=CC=C4)C=C3)=C2)SC(NC5=CC=CC=C5)=N1 | C_6_H_6_ | 4- C_7_H_7_O |
| 110 | C110 | FC1=C(OC(F)(F)F)C=CC(C2=CC(C3=CN=C(NC4=CC=CC=C4)S3)=NO2)=C1 | C_6_H_6_ | 3-F,4-OCF_3_ |
| 111 | C111 | CC1=C(C2=NOC(C3=C(F)C=C(N4CCCOC4)C=C3)=C2)SC(NC5=CC=CC=C5)=N1 | C_6_H_6_ | 2-F,4-C_4_H_8_NO |
| 112 | C112 | FC1=C(S(C)(=O)=O)C=CC(C2=CC(C3=CN=C(NC4=CC=CC=C4)S3)=NO2)=C1 | C_6_H_6_ | 3-F, 4-CH_3_O_2_S |
| 113 | C113 | FC1=CC=C(N2N=CC=C2)C(C3=CC(C4=CN=C(NC5=CC=CC=C5)S4)=NO3)=C1 | C_6_H_6_ | 2-C_3_H_3_N_2_,5-F |
| 114 | C114 | CC1=C(C2=NOC(C3=CC=CC=C3C4=CN=CN=C4)=C2)SC(NC5=CC=CC=C5)=N1 | C_6_H_6_ | 2-C_4_H_4_N_2_ |
| 115 | C115 | CC1=C(C2=NOC(C3=C(C)C=C(N4CCCC4)C(F)=C3)=C2)SC(NC5=CC=CC=C5)=N1 | C_6_H_6_ | 2-CH_3_ ,4-C_4_H_8_N,5-F |
| 116 | C116 | COC1=CC=CC(C2=CC(C3=CN=C(NC4=CC=CC=C4)S3)=NO2)=C1 | C_6_H_6_ | 3-OCH_3_ |
| 117 | C117 | CC1=C(C2=NOC(C3=C(O)C=CC([N+]([O-])=O)=C3)=C2)SC(NC4=CC=CC=C4)=N1 | C_6_H_6_ | 2-OH, 5-NO_2_ |
| 118 | C118 | CC1=C(C2=NOC(C3=C([N+]([O-])=O)C=CC(O)=C3)=C2)SC(NC4=CC=CC=C4)=N1 | C_6_H_6_ | 2- NO_2_, 5-OH |
| 119 | C119 | CC1=C(C2=NOC(C3=C([N+]([O-])=O)C=CC(Cl)=C3)=C2)SC(NC4=CC=CC=C4)=N1 | C_6_H_6_ | 2- NO_2_,5-Cl |
| 120 | C120 | CC1=C(C2=NOC(C3=C(OC)C=C([N+]([O-])=O)C=C3)=C2)SC(NC4=CC=CC=C4)=N1 | C_6_H_6_ | 2-OCH_3_,4-NO_2_ |
| 121 | C121 | CC1=C(C2=NOC(C3=C([N+]([O-])=O)C(OC)=CC=C3)=C2)SC(NC4=CC=CC=C4)=N1 | C_6_H_6_ | 2-NO_2_ ,3-OCH_3_, |
| 122 | C122 | ClC(C=CC([N+]([O-])=O)=C1)=C1C2=CC(C3=CN=C(NC4=CC=CC=C4)S3)=NO2 | C_6_H_6_ | 2-Cl, 5-NO_2_ |
| 123 | C123 | COC1=CC(C2=CC(C3=CN=C(NC4=CC=CC=C4)S3)=NO2)=C([N+]([O-])=O)C=C1 | C_6_H_6_ | 2- NO_2,_ 5-OCH_3_ |
| 124 | C124 | OC(C([N+]([O-])=O)=CC=C1)=C1C2=CC(C3=CN=C(NC4=CC=CC=C4)S3)=NO2 | C_6_H_6_ | 2-OH, 3-NO_2_ |
| 125 | C125 | OC(C([N+]([O-])=O)=CC(OC)=C1)=C1C2=CC(C3=CN=C(NC4=CC=CC=C4)S3)=NO2 | C_6_H_6_ | 2-OH,3-NO_2_,5-OCH_3_ |
| 126 | C126 | COC1=CC=C([N+]([O-])=O)C(C2=CC(C3=CN=C(NC4=CC=CC=C4)S3)=NO2)=C1[N+]([O-])=O | C_6_H_6_ | 2,6-NO_2_, 3-OCH_3_ |
| 127 | C127 | ClC1=CC(Cl)=CC(C2=CC(C3=CN=C(NC4=CC=CC=C4)S3)=NO2)=C1Cl | C_6_H_6_ | 2,3,5-Cl |
| 128 | C128 | CC1=C(C2=NOC(C3=CC(OC)=C(F)C=C3)=C2)SC(NC4=CC=CC=C4)=N1 | C_6_H_6_ | 3-OCH_3_, 4-F |
| 129 | C129 | IC1=C(OC)C=CC(C2=CC(C3=CN=C(NC4=CC=CC=C4)S3)=NO2)=C1 | C_6_H_6_ | 3-I, 4-OCH_3_ |
| 130 | C130 | ClC1=CC=C(OC)C(Cl)=C1C2=CC(C3=CN=C(NC4=CC=CC=C4)S3)=NO2 | C_6_H_6_ | 2,6-Cl, 3-OCH_3_ |
| 131 | C131 | COC(C=C1)=C(OC(C)C)C=C1C2=CC(C3=CN=C(NC4=CC=CC=C4)S3)=NO2 | C_6_H_6_ | 3-C_3_H_7_O, 4-OCH_3_ |
| 132 | C132 | COC1=CC=CC(C2=CC(C3=CN=C(NC4=CC=CC=C4)S3)=NO2)=C1OCC5=CC=CC(Cl)=C5 | C_6_H_6_ | 2-C_7_H_6_ClO, 3-OCH_3_ |
| 133 | C133 | COC(C=C1)=C(OCC)C=C1C2=CC(C3=CN=C(NC4=CC=CC=C4)S3)=NO2 | C_6_H_6_ | 3-OC_2_H_5_ , 4-OCH_3_ |
| 134 | C134 | CC1=C(C2=NOC(C3=CC(F)=C(O)C(OC)=C3)=C2)SC(NC4=CC=CC=C4)=N1 | C_6_H_6_ | 3-F, 4-OH, 5-OCH_3_ |
| 135 | C135 | CC1=C(C2=NOC(C3=CC(F)=C(OC)C=C3)=C2)SC(NC4=CC=CC=C4)=N1 | C_6_H_6_ | 3-F, 4- OCH_3_ |
| 136 | C136 | CC1=C(C2=NOC(C3=CC(Cl)=C(OC)C=C3)=C2)SC(NC4=CC=CC=C4)=N1 | C_6_H_6_ | 3-Cl, 4- OCH_3_ |
| 137 | C137 | CC1=C(C2=NOC(C3=C(Br)C(O)=C(OC)C=C3)=C2)SC(NC4=CC=CC=C4)=N1 | C_6_H_6_ | 2-Br, 3-OH, 4- OCH_3_ |
| 138 | C138 | CC1=C(C2=NOC(C3=CC(OC)=C(OCC4=CC=CC=C4)C=C3)=C2)SC(NC5=CC=CC=C5)=N1 | C_6_H_6_ | 3- OCH_3_ ,4-C_7_H_7_O |
| 139 | C139 | CC1=C(C2=NOC(C3=CC(Br)=C(OC(C)C)C(OC)=C3)=C2)SC(NC4=CC=CC=C4)=N1 | C_6_H_6_ | 3-Br, 4-C_3_H_7_O, 5-OCH_3_ |
| 140 | C140 | CC1=C(C2=NOC(C3=C(OCC4=CC(Cl)=C(Cl)C=C4)C(OC)=CC=C3)=C2)SC(NC5=CC=CC=C5)=N1 | C_6_H_6_ | 2-C_7_H_5_Cl_2_O, 3-OCH_3_ |
| 141 | C141 | CC1=C(C2=NOC(C3=CC=CC=C3)=C2)SC(NC4=CC=C(Cl)C=C4)=N1 | C_6_H_5_Cl | H |
| 142 | C142 | CC1=C(C2=NOC(C3=C(O)C=CC=C3)=C2)SC(NC4=CC=C(Cl)C=C4)=N1 | C_6_H_5_Cl | 2-OH |
| 143 | C143 | CC1=C(C2=NOC(C3=C(Cl)C=CC=C3)=C2)SC(NC4=CC=C(Cl)C=C4)=N1 | C_6_H_5_Cl | 2-Cl |
| 144 | C144 | CC1=C(C2=NOC(C3=CC(Cl)=CC=C3)=C2)SC(NC4=CC=C(Cl)C=C4)=N1 | C_6_H_5_Cl | 3-Cl |
| 145 | C145 | CC1=C(C2=NOC(C3=CC=C(Cl)C=C3)=C2)SC(NC4=CC=C(Cl)C=C4)=N1 | C_6_H_5_Cl | 4-Cl |
| 146 | C146 | CC1=C(C2=NOC(C3=C(Cl)C=C(Cl)C=C3)=C2)SC(NC4=CC=C(Cl)C=C4)=N1 | C_6_H_5_Cl | 2,4-Cl |
| 147 | C147 | ClC1=CC=CC(Cl)=C1C2=CC(C3=CN=C(NC4=CC=C(Cl)C=C4)S3)=NO2 | C_6_H_5_Cl | 2,6-Cl |
| 148 | C148 | ClC(C(Cl)=CC=C1)=C1C2=CC(C3=CN=C(NC4=CC=C(Cl)C=C4)S3)=NO2 | C_6_H_5_Cl | 2,3-Cl |
| 149 | C149 | ClC1=CC(Cl)=CC(C2=CC(C3=CN=C(NC4=CC=C(Cl)C=C4)S3)=NO2)=C1 | C_6_H_5_Cl | 3,5-Cl |
| 150 | C150 | ClC1=C(Cl)C=CC(C2=CC(C3=CN=C(NC4=CC=C(Cl)C=C4)S3)=NO2)=C1 | C_6_H_5_Cl | 3,4-Cl |
| 151 | C151 | ClC(C=C1)=CC=C1NC2=NC=C(C3=NOC(C4=CC([N+]([O-])=O)=CC=C4)=C3)S2 | C_6_H_5_Cl | 3-NO_2_ |
| 152 | C152 | ClC(C=C1)=CC=C1NC2=NC=C(C3=NOC(C4=CC=C([N+]([O-])=O)C=C4)=C3)S2 | C_6_H_5_Cl | 4-NO_2_ |
| 153 | C153 | ClC(C=C1)=CC=C1NC2=NC=C(C3=NOC(C4=C([N+]([O-])=O)C=CC=C4)=C3)S2 | C_6_H_5_Cl | 2-NO_2_ |
| 154 | C154 | ClC(C=C1)=CC=C1NC2=NC=C(C3=NOC(C4=C([N+]([O-])=O)C=C([N+]([O-])=O)C=C4)=C3)S2 | C_6_H_5_Cl | 2,4-NO_2_ |
| 155 | C155 | ClC(C=C1)=CC=C1NC2=NC=C(C3=NOC(C4=C([N+]([O-])=O)C=CC=C4[N+]([O-])=O)=C3)S2 | C_6_H_5_Cl | 2,6-NO_2_ |
| 156 | C156 | ClC(C=C1)=CC=C1NC2=NC=C(C3=NOC(C4=CC([N+]([O-])=O)=CC([N+]([O-])=O)=C4)=C3)S2 | C_6_H_5_Cl | 3,5-NO_2_ |
| 157 | C157 | CC1=C(C)C(C)=CC(C2=CC(C3=CN=C(NC4=CC=C(Cl)C=C4)S3)=NO2)=C1 | C_6_H_5_Cl | 3,4,5-CH_3_ |
| 158 | C158 | ClC(C=C1)=CC=C1NC2=NC=C(C3=NOC(C4=C(OC)C=CC=C4)=C3)S2 | C_6_H_5_Cl | 2-OCH_3_ |
| 159 | C159 | ClC(C=C1)=CC=C1NC2=NC=C(C3=NOC(C4=CC=C(OC)C=C4)=C3)S2 | C_6_H_5_Cl | 4-OCH_3_ |
| 160 | C160 | ClC(C=C1)=CC=C1NC2=NC=C(C3=NOC(C4=C(OC)C=CC(OC)=C4)=C3)S2 | C_6_H_5_Cl | 2,5-OCH_3_ |
| 161 | C161 | ClC(C=C1)=CC=C1NC2=NC=C(C3=NOC(C4=CC(OC)=C(OC)C=C4)=C3)S2 | C_6_H_5_Cl | 3,4-OCH_3_ |
| 162 | C162 | CN(C)C(C=C1)=CC=C1C2=CC(C3=CN=C(NC4=CC=C(Cl)C=C4)S3)=NO2 | C_6_H_5_Cl | 4-N(CH_3_)_2_ |
| 163 | C163 | FC(C=C1)=CC=C1C2=CC(C3=CN=C(NC4=CC=C(Cl)C=C4)S3)=NO2 | C_6_H_5_Cl | 4-F |
| 164 | C164 | BrC(C=C1)=CC=C1C2=CC(C3=CN=C(NC4=CC=C(Cl)C=C4)S3)=NO2 | C_6_H_5_Cl | 4-Br |
| 165 | C165 | CC1=C(C2=NOC(C3=CC=C(O)C=C3)=C2)SC(NC4=CC=C(Cl)C=C4)=N1 | C_6_H_5_Cl | 4-OH |
| 166 | C166 | ClC(C=C1)=CC=C1NC2=NC=C(C3=NOC(C4=CC(OC)=C(OC)C(OC)=C4)=C3)S2 | C_6_H_5_Cl | 3,4,5-OCH_3_ |
| 167 | C167 | CC1=C(C2=NOC(C3=CC(OC)=C(O)C=C3)=C2)SC(NC4=CC=C(Cl)C=C4)=N1 | C_6_H_5_Cl | 3-OCH_3_,4-Cl |
| 168 | C168 | CC1=C(C2=NOC(C3=C(Cl)C=CC(C(F)(F)F)=C3)=C2)SC(NC4=CC=C(Cl)C=C4)=N1 | C_6_H_5_Cl | 2-Cl, 5-CF_3_ |
| 169 | C169 | CC1=C(C2=NOC(C3=CC=C(C(F)(F)F)C=C3)=C2)SC(NC4=CC=C(Cl)C=C4)=N1 | C_6_H_5_Cl | 4-CF_3_ |
| 170 | C170 | CC1=C(C2=NOC(C3=CC=C(C(F)(F)F)C=C3)=C2)SC(NC4=CC=C(Cl)C=C4)=N1 | C_6_H_5_Cl | 2-CF_3_ |
| 171 | C171 | CC1=C(C2=NOC(C3=C(Cl)C=C(N)C=C3)=C2)SC(NC4=CC=C(Cl)C=C4)=N1 | C_6_H_5_Cl | 2-Cl,4-NH_3_ |
| 172 | C172 | CC1=C(C2=NOC(C3=CC(OC)=C(O)C([N+]([O-])=O)=C3)=C2)SC(NC4=CC=C(Cl)C=C4)=N1 | C_6_H_5_Cl | 3-NO_2_,4-OH,5- OCH_3_ |
| 173 | C173 | BrC(C=C(O)C(OC)=C1)=C1C2=CC(C3=CN=C(NC4=CC=C(Cl)C=C4)S3)=NO2 | C_6_H_5_Cl | 2-Br,4-OH,5- OCH_3_ |
| 174 | C174 | CC1=C(C2=NOC(C3=CC(OC)=C(O)C(Br)=C3)=C2)SC(NC4=CC=C(Cl)C=C4)=N1 | C_6_H_5_Cl | 3-Br,4-OH,5- OCH_3_ |
| 175 | C175 | OC(C(OCC)=C1)=C(Br)C=C1C2=CC(C3=CN=C(NC4=CC=C(Cl)C=C4)S3)=NO2 | C_6_H_5_Cl | 3-Br,4-OH 5-C_2_H_5_O |
| 176 | C176 | CC1=C(C2=NOC(C3=CC(OC)=C(O)C(I)=C3)=C2)SC(NC4=CC=C(Cl)C=C4)=N1 | C_6_H_5_Cl | 3- OCH_3_,4-OH,5-I |
| 177 | C177 | BrC1=C(OCCCCC)C(OC)=CC(C2=CC(C3=CN=C(NC4=CC=C(Cl)C=C4)S3)=NO2)=C1 | C_6_H_5_Cl | 3-Br,4-C_5_H_12_O,5-OCH_3_ |
| 178 | C178 | COC(C=C1)=C(OCC2=CC=CC=C2)C(Br)=C1C3=CC(C4=CN=C(NC5=CC=C(Cl)C=C5)S4)=NO3 | C_6_H_5_Cl | 2-Br,3-C_7_H_7_O,4-OCH_3_ |
| 179 | C179 | CC1=C(C2=NOC(C3=CC=C(OCC4=CC=CC=C4)C=C3)=C2)SC(NC5=CC=C(Cl)C=C5)=N1 | C_6_H_5_Cl | 4- C_7_H_7_O |
| 180 | C180 | FC1=C(OC(F)(F)F)C=CC(C2=CC(C3=CN=C(NC4=CC=C(Cl)C=C4)S3)=NO2)=C1 | C_6_H_5_Cl | 3-F,4-OCF_3_ |
| 181 | C181 | CC1=C(C2=NOC(C3=C(F)C=C(N4CCCOC4)C=C3)=C2)SC(NC5=CC=C(Cl)C=C5)=N1 | C_6_H_5_Cl | 2-F,4-C_4_H_8_NO |
| 182 | C182 | ClC(C=C1)=CC=C1NC2=NC=C(C3=NOC(C4=CC(F)=C(S(C)(=O)=O)C=C4)=C3)S2 | C_6_H_5_Cl | 3-F, 4-CH_3_O_2_S |
| 183 | C183 | ClC(C=C1)=CC=C1NC2=NC=C(C3=NOC(C4=CC(F)=CC=C4N5N=CC=C5)=C3)S2 | C_6_H_5_Cl | 2-C_3_H_3_N_2_,5-F |
| 184 | C184 | CC1=C(C2=NOC(C3=CC=CC=C3C4=CN=CN=C4)=C2)SC(NC5=CC=C(Cl)C=C5)=N1 | C_6_H_5_Cl | 2-C_4_H_4_N_2_ |
| 185 | C185 | CC1=C(C2=NOC(C3=C(C)C=C(N4CCCC4)C(F)=C3)=C2)SC(NC5=CC=C(Cl)C=C5)=N1 | C_6_H_5_Cl | 2-CH_3_ ,4-C_4_H_8_N,5-F |
| 186 | C186 | ClC(C=C1)=CC=C1NC2=NC=C(C3=NOC(C4=CC(OC)=CC=C4)=C3)S2 | C_6_H_5_Cl | 3-OCH_3_ |
| 187 | C187 | CC1=C(C2=NOC(C3=C(O)C=CC([N+]([O-])=O)=C3)=C2)SC(NC4=CC=C(Cl)C=C4)=N1 | C_6_H_5_Cl | 2-OH, 5-NO_2_ |
| 188 | C188 | CC1=C(C2=NOC(C3=C([N+]([O-])=O)C=CC(O)=C3)=C2)SC(NC4=CC=C(Cl)C=C4)=N1 | C_6_H_5_Cl | 2- NO_2_, 5-OH |
| 189 | C189 | CC1=C(C2=NOC(C3=C([N+]([O-])=O)C=CC(Cl)=C3)=C2)SC(NC4=CC=C(Cl)C=C4)=N1 | C_6_H_5_Cl | 2- NO_2_,5-Cl |
| 190 | C190 | CC1=C(C2=NOC(C3=C(OC)C=C([N+]([O-])=O)C=C3)=C2)SC(NC4=CC=C(Cl)C=C4)=N1 | C_6_H_5_Cl | 2-OCH_3_,4-NO_2_ |
| 191 | C191 | CC1=C(C2=NOC(C3=C([N+]([O-])=O)C(OC)=CC=C3)=C2)SC(NC4=CC=C(Cl)C=C4)=N1 | C_6_H_5_Cl | 2-NO_2_ ,3-OCH_3_, |
| 192 | C192 | ClC(C=CC([N+]([O-])=O)=C1)=C1C2=CC(C3=CN=C(NC4=CC=C(Cl)C=C4)S3)=NO2 | C_6_H_5_Cl | 2-Cl, 5-NO_2_ |
| 193 | C193 | COC1=CC(C2=CC(C3=CN=C(NC4=CC=C(Cl)C=C4)S3)=NO2)=C([N+]([O-])=O)C=C1 | C_6_H_5_Cl | 2- NO_2,_ 5-OCH_3_ |
| 194 | C194 | OC(C([N+]([O-])=O)=CC=C1)=C1C2=CC(C3=CN=C(NC4=CC=C(Cl)C=C4)S3)=NO2 | C_6_H_5_Cl | 2-OH, 3-NO_2_ |
| 195 | C195 | OC(C([N+]([O-])=O)=CC(OC)=C1)=C1C2=CC(C3=CN=C(NC4=CC=C(Cl)C=C4)S3)=NO2 | C_6_H_5_Cl | 2-OH,3-NO_2_,5-OCH_3_ |
| 196 | C196 | COC1=CC=C([N+]([O-])=O)C(C2=CC(C3=CN=C(NC4=CC=C(Cl)C=C4)S3)=NO2)=C1[N+]([O-])=O | C_6_H_5_Cl | 2,6-NO_2_, 3-OCH_3_ |
| 197 | C197 | ClC1=CC(Cl)=CC(C2=CC(C3=CN=C(NC4=CC=C(Cl)C=C4)S3)=NO2)=C1Cl | C_6_H_5_Cl | 2,3,5-Cl |
| 198 | C198 | CC1=C(C2=NOC(C3=CC(OC)=C(F)C=C3)=C2)SC(NC4=CC=C(Cl)C=C4)=N1 | C_6_H_5_Cl | 3-OCH_3_, 4-F |
| 199 | C199 | IC1=C(OC)C=CC(C2=CC(C3=CN=C(NC4=CC=C(Cl)C=C4)S3)=NO2)=C1 | C_6_H_5_Cl | 3-I, 4-OCH_3_ |
| 200 | C200 | ClC1=CC=C(OC)C(Cl)=C1C2=CC(C3=CN=C(NC4=CC=C(Cl)C=C4)S3)=NO2 | C_6_H_5_Cl | 2,6-Cl, 3-OCH_3_ |
| 201 | C201 | COC(C=C1)=C(OC(C)C)C=C1C2=CC(C3=CN=C(NC4=CC=C(Cl)C=C4)S3)=NO2 | C_6_H_5_Cl | 3-C_3_H_7_O, 4-OCH_3_ |
| 202 | C202 | ClC(C=C1)=CC=C1NC2=NC=C(C3=NOC(C4=C(OCC5=CC=CC(Cl)=C5)C(OC)=CC=C4)=C3)S2 | C_6_H_5_Cl | 2-C_7_H_6_ClO, 3-OCH_3_ |
| 203 | C203 | ClC(C=C1)=CC=C1NC2=NC=C(C3=NOC(C4=CC(OCC)=C(OC)C=C4)=C3)S2 | C_6_H_5_Cl | 3-OC_2_H_5_ , 4-OCH_3_ |
| 204 | C204 | CC1=C(C2=NOC(C3=CC(F)=C(O)C(OC)=C3)=C2)SC(NC4=CC=C(Cl)C=C4)=N1 | C_6_H_5_Cl | 3-F, 4-OH, 5-OCH_3_ |
| 205 | C205 | CC1=C(C2=NOC(C3=CC(F)=C(OC)C=C3)=C2)SC(NC4=CC=C(Cl)C=C4)=N1 | C_6_H_5_Cl | 3-F, 4- OCH_3_ |
| 206 | C206 | CC1=C(C2=NOC(C3=CC(Cl)=C(OC)C=C3)=C2)SC(NC4=CC=C(Cl)C=C4)=N1 | C_6_H_5_Cl | 3-Cl, 4- OCH_3_ |
| 207 | C207 | CC1=C(C2=NOC(C3=C(Br)C(O)=C(OC)C=C3)=C2)SC(NC4=CC=C(Cl)C=C4)=N1 | C_6_H_5_Cl | 2-Br, 3-OH, 4- OCH_3_ |
| 208 | C208 | CC1=C(C2=NOC(C3=CC(OC)=C(OCC4=CC=CC=C4)C=C3)=C2)SC(NC5=CC=C(Cl)C=C5)=N1 | C_6_H_5_Cl | 3- OCH_3_ ,4-C_7_H_7_O |
| 209 | C209 | CC1=C(C2=NOC(C3=CC(Br)=C(OC(C)C)C(OC)=C3)=C2)SC(NC4=CC=C(Cl)C=C4)=N1 | C_6_H_5_Cl | 3-Br, 4-C_3_H_7_O, 5-OCH_3_ |
| 210 | C210 | CC1=C(C2=NOC(C3=C(OCC4=CC(Cl)=C(Cl)C=C4)C(OC)=CC=C3)=C2)SC(NC5=CC=C(Cl)C=C5)=N1 | C_6_H_5_Cl | 2-C_7_H_5_Cl_2_O, 3-OCH_3_ |
