# Supplementary material for: Design and in silico evaluation of Thiazole–Isoxazole hybrids as PPARγ-Targeted antidiabetic agents
Source: Front Bioinform. 2026 Aug 11;6:1873262. doi: 10.3389/fbinf.2026.1873262 (PMC13503372; doi:10.3389/fbinf.2026.1873262)
Supplement: Supplementary file 2 [file Table2.docx]

**Supplementary file 2**

**Table 1: Designed ligands with docking score (**kcal/mol)

| Sl no: | Compound code | Canonical smile | Docking score |  |
| --- | --- | --- | --- | --- |
| 1 | C1 | CNC1=NC(C)=C(C2=NOC(C3=CC=CC=C3)=C2)S1 | -7.603 |  |
| 2 | C2 | CNC1=NC(C)=C(C2=NOC(C3=C(O)C=CC=C3)=C2)S1 | -7.544 |  |
| 3 | C3 | CNC1=NC(C)=C(C2=NOC(C3=C(Cl)C=CC=C3)=C2)S1 | -8.962 |  |
| 4 | C4 | CNC1=NC(C)=C(C2=NOC(C3=CC(Cl)=CC=C3)=C2)S1 | -7.418 |  |
| 5 | C5 | CNC1=NC(C)=C(C2=NOC(C3=CC=C(Cl)C=C3)=C2)S1 | -7.587 |  |
| 6 | C6 | CNC1=NC(C)=C(C2=NOC(C3=C(Cl)C=C(Cl)C=C3)=C2)S1 | -8.946 |  |
| 7 | C7 | CNC1=NC=C(C2=NOC(C3=C(Cl)C=CC=C3Cl)=C2)S1 | -6.553 |  |
| 8 | C8 | CNC1=NC=C(C2=NOC(C3=C(Cl)C(Cl)=CC=C3)=C2)S1 | -7.938 |  |
| 9 | C9 | CNC1=NC=C(C2=NOC(C3=CC(Cl)=CC(Cl)=C3)=C2)S1 | -8.240 |  |
| 10 | C10 | CNC1=NC=C(C2=NOC(C3=CC(Cl)=C(Cl)C=C3)=C2)S1 | -7.164 |  |
| 11 | C11 | CNC1=NC=C(C2=NOC(C3=CC([N+]([O-])=O)=CC=C3)=C2)S1 | -7.773 |  |
| 12 | C12 | CNC1=NC=C(C2=NOC(C3=CC=C([N+]([O-])=O)C=C3)=C2)S1 | -7.437 |  |
| 13 | C13 | CNC1=NC=C(C2=NOC(C3=C([N+]([O-])=O)C=CC=C3)=C2)S1 | -6.843 |  |
| 14 | C14 | CNC1=NC=C(C2=NOC(C3=C([N+]([O-])=O)C=C([N+]([O-])=O)C=C3)=C2)S1 | -6.548 |  |
| 15 | C15 | CNC1=NC=C(C2=NOC(C3=C([N+]([O-])=O)C=CC=C3[N+]([O-])=O)=C2)S1 | -6.125 |  |
| 16 | C16 | CNC1=NC=C(C2=NOC(C3=CC([N+]([O-])=O)=CC([N+]([O-])=O)=C3)=C2)S1 | -7.568 |  |
| 17 | C17 | CNC1=NC=C(C2=NOC(C3=CC(C)=C(C)C(C)=C3)=C2)S1 | -5.923 |  |
| 18 | C18 | CNC1=NC=C(C2=NOC(C3=C(OC)C=CC=C3)=C2)S1 | -7.661 |  |
| 19 | C19 | CNC1=NC=C(C2=NOC(C3=CC=C(OC)C=C3)=C2)S1 | -7.208 |  |
| 20 | C20 | CNC1=NC=C(C2=NOC(C3=C(OC)C=CC(OC)=C3)=C2)S1 | -6.443 |  |
| 21 | C21 | CNC1=NC=C(C2=NOC(C3=CC(OC)=C(OC)C=C3)=C2)S1 | -6.398 |  |
| 22 | C22 | CNC1=NC=C(C2=NOC(C3=CC=C(N(C)C)C=C3)=C2)S1 | -5.808 |  |
| 23 | C23 | CNC1=NC=C(C2=NOC(C3=CC=C(F)C=C3)=C2)S1 | -8.074 |  |
| 24 | C24 | CNC1=NC=C(C2=NOC(C3=CC=C(Br)C=C3)=C2)S1 | -7.309 |  |
| 25 | C25 | CNC1=NC(C)=C(C2=NOC(C3=CC=C(O)C=C3)=C2)S1 | -8.688 |  |
| 26 | C26 | CNC1=NC=C(C2=NOC(C3=CC(OC)=C(OC)C(OC)=C3)=C2)S1 | -5.967 |  |
| 27 | C27 | CNC1=NC(C)=C(C2=NOC(C3=CC(OC)=C(O)C=C3)=C2)S1 | -9.398 |  |
| 28 | C28 | CNC1=NC(C)=C(C2=NOC(C3=C(Cl)C=CC(C(F)(F)F)=C3)=C2)S1 | -7.443 |  |
| 29 | C29 | CNC1=NC(C)=C(C2=NOC(C3=CC=C(C(F)(F)F)C=C3)=C2)S1 | -9.970 |  |
| 30 | C30 | CNC1=NC(C)=C(C2=NOC(C3=C(C(F)(F)F)C=CC=C3)=C2)S1 | -8.858 |  |
| 31 | C31 | CNC1=NC(C)=C(C2=NOC(C3=C(Cl)C=C(N)C=C3)=C2)S1 | -8.508 |  |
| 32 | C32 | CNC1=NC(C)=C(C2=NOC(C3=CC(OC)=C(O)C([N+]([O-])=O)=C3)=C2)S1 | -6.172 |  |
| 33 | C33 | CNC1=NC=C(C2=NOC(C3=C(Br)C=C(O)C(OC)=C3)=C2)S1 | -6.923 |  |
| 34 | C34 | CNC1=NC(C)=C(C2=NOC(C3=CC(OC)=C(O)C(Br)=C3)=C2)S1 | -6.938 |  |
| 35 | C35 | CNC1=NC=C(C2=NOC(C3=CC(Br)=C(O)C(OCC)=C3)=C2)S1 | -6.900 |  |
| 36 | C36 | CNC1=NC(C)=C(C2=NOC(C3=CC(OC)=C(O)C(I)=C3)=C2)S1 | -7.035 |  |
| 37 | C37 | CNC1=NC=C(C2=NOC(C3=CC(Br)=C(OCCCCC)C(OC)=C3)=C2)S1 | -9.998 |  |
| 38 | C38 | CNC1=NC=C(C2=NOC(C3=C(Br)C(OCC4=CC=CC=C4)=C(OC)C=C3)=C2)S1 | -9.309 |  |
| 39 | C39 | CNC1=NC(C)=C(C2=NOC(C3=CC=C(OCC4=CC=CC=C4)C=C3)=C2)S1 | -8.343 |  |
| 40 | C40 | CNC1=NC=C(C2=NOC(C3=CC(F)=C(OC(F)(F)F)C=C3)=C2)S1 | -8.125 |  |
| 41 | C41 | CNC1=NC(C)=C(C2=NOC(C3=C(F)C=C(N4CCCOC4)C=C3)=C2)S1 | -6.428 |  |
| 42 | C42 | CNC1=NC=C(C2=NOC(C3=CC(F)=C(S(C)(=O)=O)C=C3)=C2)S1 | -6.109 |  |
| 43 | C43 | CNC1=NC=C(C2=NOC(C3=CC(F)=CC=C3N4N=CC=C4)=C2)S1 | -8.037 |  |
| 44 | C44 | CNC1=NC(C)=C(C2=NOC(C3=CC=CC=C3C4=CN=CN=C4)=C2)S1 | -9.175 |  |
| 45 | C45 | CNC1=NC(C)=C(C2=NOC(C3=C(C)C=C(N4CCCC4)C(F)=C3)=C2)S1 | -6.456 |  |
| 46 | C46 | CNC1=NC=C(C2=NOC(C3=CC(OC)=CC=C3)=C2)S1 | -7.040 |  |
| 47 | C47 | CNC1=NC(C)=C(C2=NOC(C3=C(O)C=CC([N+]([O-])=O)=C3)=C2)S1 | -8.051 |  |
| 48 | C48 | CNC1=NC(C)=C(C2=NOC(C3=C([N+]([O-])=O)C=CC(O)=C3)=C2)S1 | -6.608 |  |
| 49 | C49 | CNC1=NC(C)=C(C2=NOC(C3=C([N+]([O-])=O)C=CC(Cl)=C3)=C2)S1 | -6.953 |  |
| 50 | C50 | CNC1=NC(C)=C(C2=NOC(C3=C(OC)C=C([N+]([O-])=O)C=C3)=C2)S1 | -6.950 |  |
| 51 | C51 | CNC1=NC(C)=C(C2=NOC(C3=C([N+]([O-])=O)C(OC)=CC=C3)=C2)S1 | -7.109 |  |
| 52 | C52 | CNC1=NC=C(C2=NOC(C3=C(Cl)C=CC([N+]([O-])=O)=C3)=C2)S1 | -5.966 |  |
| 53 | C53 | CNC1=NC=C(C2=NOC(C3=C([N+]([O-])=O)C=CC(OC)=C3)=C2)S1 | -5.640 |  |
| 54 | C54 | CNC1=NC=C(C2=NOC(C3=C(O)C([N+]([O-])=O)=CC=C3)=C2)S1 | -7.909 |  |
| 55 | C55 | CNC1=NC=C(C2=NOC(C3=C(O)C([N+]([O-])=O)=CC(OC)=C3)=C2)S1 | -6.249 |  |
| 56 | C56 | CNC1=NC=C(C2=NOC(C3=C([N+]([O-])=O)C(OC)=CC=C3[N+]([O-])=O)=C2)S1 | -5.464 |  |
| 57 | C57 | CNC1=NC=C(C2=NOC(C3=C(Cl)C(Cl)=CC(Cl)=C3)=C2)S1 | -7.482 |  |
| 58 | C58 | CNC1=NC(C)=C(C2=NOC(C3=CC(OC)=C(F)C=C3)=C2)S1 | -9.455 |  |
| 59 | C59 | IC1=C(OC)C=CC(C2=CC(C3=CN=C(NC)S3)=NO2)=C1 | -6.518 |  |
| 60 | C60 | CNC1=NC=C(C2=NOC(C3=C(Cl)C(OC)=CC=C3Cl)=C2)S1 | -6.763 |  |
| 61 | C61 | CNC1=NC=C(C2=NOC(C3=CC(OC(C)C)=C(OC)C=C3)=C2)S1 | -6.615 |  |
| 62 | C62 | CNC1=NC=C(C2=NOC(C3=C(OCC4=CC=CC(Cl)=C4)C(OC)=CC=C3)=C2)S1 | -9.781 |  |
| 63 | C63 | CNC1=NC=C(C2=NOC(C3=CC(OCC)=C(OC)C=C3)=C2)S1 | -6.958 |  |
| 64 | C64 | CNC1=NC(C)=C(C2=NOC(C3=CC(F)=C(O)C(OC)=C3)=C2)S1 | -9.511 |  |
| 65 | C65 | CNC1=NC(C)=C(C2=NOC(C3=CC(F)=C(OC)C=C3)=C2)S1 | -7.133 |  |
| 66 | C66 | CNC1=NC(C)=C(C2=NOC(C3=CC(Cl)=C(OC)C=C3)=C2)S1 | -8.046 |  |
| 67 | C67 | CNC1=NC(C)=C(C2=NOC(C3=C(Br)C(O)=C(OC)C=C3)=C2)S1 | -7.270 |  |
| 68 | C68 | CNC1=NC(C)=C(C2=NOC(C3=CC(OC)=C(OCC4=CC=CC=C4)C=C3)=C2)S1 | -8.609 |  |
| 69 | C69 | CNC1=NC(C)=C(C2=NOC(C3=CC(Br)=C(OC(C)C)C(OC)=C3)=C2)S1 | -7.088 |  |
| 70 | C70 | CNC1=NC(C)=C(C2=NOC(C3=C(OCC4=CC(Cl)=C(Cl)C=C4)C(OC)=CC=C3)=C2)S1 | -9.406 |  |
| 71 | C71 | CC1=C(C2=NOC(C3=CC=CC=C3)=C2)SC(NC4=CC=CC=C4)=N1 | -8.548 |  |
| 72 | C72 | CC1=C(C2=NOC(C3=C(O)C=CC=C3)=C2)SC(NC4=CC=CC=C4)=N1 | -8.029 |  |
| 73 | C73 | CC1=C(C2=NOC(C3=C(Cl)C=CC=C3)=C2)SC(NC4=CC=CC=C4)=N1 | -8.214 |  |
| 74 | C74 | CC1=C(C2=NOC(C3=CC(Cl)=CC=C3)=C2)SC(NC4=CC=CC=C4)=N1 | -7.678 |  |
| 75 | C75 | CC1=C(C2=NOC(C3=CC=C(Cl)C=C3)=C2)SC(NC4=CC=CC=C4)=N1 | -8.056 |  |
| 76 | C76 | CC1=C(C2=NOC(C3=C(Cl)C=C(Cl)C=C3)=C2)SC(NC4=CC=CC=C4)=N1 | -8.203 |  |
| 77 | C77 | ClC1=CC=CC(Cl)=C1C2=CC(C3=CN=C(NC4=CC=CC=C4)S3)=NO2 | -8.202 |  |
| 78 | C78 | ClC(C(Cl)=CC=C1)=C1C2=CC(C3=CN=C(NC4=CC=CC=C4)S3)=NO2 | -8.545 |  |
| 79 | C79 | ClC1=CC(Cl)=CC(C2=CC(C3=CN=C(NC4=CC=CC=C4)S3)=NO2)=C1 | -7.756 |  |
| 80 | C80 | ClC1=C(Cl)C=CC(C2=CC(C3=CN=C(NC4=CC=CC=C4)S3)=NO2)=C1 | -6.827 |  |
| 81 | C81 | O=[N+](C1=CC=CC(C2=CC(C3=CN=C(NC4=CC=CC=C4)S3)=NO2)=C1)[O-] | -6.764 |  |
| 82 | C82 | O=[N+](C(C=C1)=CC=C1C2=CC(C3=CN=C(NC4=CC=CC=C4)S3)=NO2)[O-] | -7.989 |  |
| 83 | C83 | O=[N+](C(C=CC=C1)=C1C2=CC(C3=CN=C(NC4=CC=CC=C4)S3)=NO2)[O-] | -7.402 |  |
| 84 | C84 | O=[N+](C(C=C([N+]([O-])=O)C=C1)=C1C2=CC(C3=CN=C(NC4=CC=CC=C4)S3)=NO2)[O-] | -6.831 |  |
| 85 | C85 | O=[N+](C(C=CC=C1[N+]([O-])=O)=C1C2=CC(C3=CN=C(NC4=CC=CC=C4)S3)=NO2)[O-] | -6.069 |  |
| 86 | C86 | O=[N+](C1=CC([N+]([O-])=O)=CC(C2=CC(C3=CN=C(NC4=CC=CC=C4)S3)=NO2)=C1)[O-] | -7.381 |  |
| 87 | C87 | CC1=C(C)C(C)=CC(C2=CC(C3=CN=C(NC4=CC=CC=C4)S3)=NO2)=C1 | -7.794 |  |
| 88 | C88 | COC(C=CC=C1)=C1C2=CC(C3=CN=C(NC4=CC=CC=C4)S3)=NO2 | -7.151 |  |
| 89 | C89 | COC(C=C1)=CC=C1C2=CC(C3=CN=C(NC4=CC=CC=C4)S3)=NO2 | -7.353 |  |
| 90 | C90 | COC(C=CC(OC)=C1)=C1C2=CC(C3=CN=C(NC4=CC=CC=C4)S3)=NO2 | -7.253 |  |
| 91 | C91 | COC1=C(OC)C=CC(C2=CC(C3=CN=C(NC4=CC=CC=C4)S3)=NO2)=C1 | -4.781 |  |
| 92 | C92 | CN(C)C(C=C1)=CC=C1C2=CC(C3=CN=C(NC4=CC=CC=C4)S3)=NO2 | -7.788 |  |
| 93 | C93 | FC(C=C1)=CC=C1C2=CC(C3=CN=C(NC4=CC=CC=C4)S3)=NO2 | -7.654 |  |
| 94 | C94 | BrC(C=C1)=CC=C1C2=CC(C3=CN=C(NC4=CC=CC=C4)S3)=NO2 | -7.872 |  |
| 95 | C95 | CC1=C(C2=NOC(C3=CC=C(O)C=C3)=C2)SC(NC4=CC=CC=C4)=N1 | -7.376 |  |
| 96 | C96 | COC1=C(OC)C(OC)=CC(C2=CC(C3=CN=C(NC4=CC=CC=C4)S3)=NO2)=C1 | -8.651 |  |
| 97 | C97 | CC1=C(C2=NOC(C3=CC(OC)=C(O)C=C3)=C2)SC(NC4=CC=CC=C4)=N1 | -7.140 |  |
| 98 | C98 | CC1=C(C2=NOC(C3=C(Cl)C=CC(C(F)(F)F)=C3)=C2)SC(NC4=CC=CC=C4)=N1 | -8.683 |  |
| 99 | C99 | CC1=C(C2=NOC(C3=CC=C(C(F)(F)F)C=C3)=C2)SC(NC4=CC=CC=C4)=N1 | -8.665 |  |
| 100 | C100 | CC1=C(C2=NOC(C3=C(C(F)(F)F)C=CC=C3)=C2)SC(NC4=CC=CC=C4)=N1 | -8.191 |  |
| 101 | C101 | CC1=C(C2=NOC(C3=C(Cl)C=C(N)C=C3)=C2)SC(NC4=CC=CC=C4)=N1 | -8.505 |  |
| 102 | C102 | CC1=C(C2=NOC(C3=CC(OC)=C(O)C([N+]([O-])=O)=C3)=C2)SC(NC4=CC=CC=C4)=N1 | -8.103 |  |
| 103 | C103 | BrC(C=C(O)C(OC)=C1)=C1C2=CC(C3=CN=C(NC4=CC=CC=C4)S3)=NO2 | -8.535 |  |
| 104 | C104 | CC1=C(C2=NOC(C3=CC(OC)=C(O)C(Br)=C3)=C2)SC(NC4=CC=CC=C4)=N1 | -7.895 |  |
| 105 | C105 | OC(C(OCC)=C1)=C(Br)C=C1C2=CC(C3=CN=C(NC4=CC=CC=C4)S3)=NO2 | -8.522 |  |
| 106 | C106 | CC1=C(C2=NOC(C3=CC(OC)=C(O)C(I)=C3)=C2)SC(NC4=CC=CC=C4)=N1 | -1.463 |  |
| 107 | C107 | BrC1=C(OCCCCC)C(OC)=CC(C2=CC(C3=CN=C(NC4=CC=CC=C4)S3)=NO2)=C1 | -10.273 |  |
| 108 | C108 | COC(C=C1)=C(OCC2=CC=CC=C2)C(Br)=C1C3=CC(C4=CN=C(NC5=CC=CC=C5)S4)=NO3 | -8.996 |  |
| 109 | C109 | CC1=C(C2=NOC(C3=CC=C(OCC4=CC=CC=C4)C=C3)=C2)SC(NC5=CC=CC=C5)=N1 | -7.848 |  |
| 110 | C110 | FC1=C(OC(F)(F)F)C=CC(C2=CC(C3=CN=C(NC4=CC=CC=C4)S3)=NO2)=C1 | -7.015 |  |
| 111 | C111 | CC1=C(C2=NOC(C3=C(F)C=C(N4CCCOC4)C=C3)=C2)SC(NC5=CC=CC=C5)=N1 | -7.758 |  |
| 112 | C112 | FC1=C(S(C)(=O)=O)C=CC(C2=CC(C3=CN=C(NC4=CC=CC=C4)S3)=NO2)=C1 | -9.264 |  |
| 113 | C113 | FC1=CC=C(N2N=CC=C2)C(C3=CC(C4=CN=C(NC5=CC=CC=C5)S4)=NO3)=C1 | -10.163 |  |
| 114 | C114 | CC1=C(C2=NOC(C3=CC=CC=C3C4=CN=CN=C4)=C2)SC(NC5=CC=CC=C5)=N1 | -7.553 |  |
| 115 | C115 | CC1=C(C2=NOC(C3=C(C)C=C(N4CCCC4)C(F)=C3)=C2)SC(NC5=CC=CC=C5)=N1 | -8.135 |  |
| 116 | C116 | COC1=CC=CC(C2=CC(C3=CN=C(NC4=CC=CC=C4)S3)=NO2)=C1 | -7.280 |  |
| 117 | C117 | CC1=C(C2=NOC(C3=C(O)C=CC([N+]([O-])=O)=C3)=C2)SC(NC4=CC=CC=C4)=N1 | -8.194 |  |
| 118 | C118 | CC1=C(C2=NOC(C3=C([N+]([O-])=O)C=CC(O)=C3)=C2)SC(NC4=CC=CC=C4)=N1 | -7.736 |  |
| 119 | C119 | CC1=C(C2=NOC(C3=C([N+]([O-])=O)C=CC(Cl)=C3)=C2)SC(NC4=CC=CC=C4)=N1 | -7.265 |  |
| 120 | C120 | CC1=C(C2=NOC(C3=C(OC)C=C([N+]([O-])=O)C=C3)=C2)SC(NC4=CC=CC=C4)=N1 | -7.158 |  |
| 121 | C121 | CC1=C(C2=NOC(C3=C([N+]([O-])=O)C(OC)=CC=C3)=C2)SC(NC4=CC=CC=C4)=N1 | -6.979 |  |
| 122 | C122 | ClC(C=CC([N+]([O-])=O)=C1)=C1C2=CC(C3=CN=C(NC4=CC=CC=C4)S3)=NO2 | -6.979 |  |
| 123 | C123 | COC1=CC(C2=CC(C3=CN=C(NC4=CC=CC=C4)S3)=NO2)=C([N+]([O-])=O)C=C1 | -7.016 |  |
| 124 | C124 | OC(C([N+]([O-])=O)=CC=C1)=C1C2=CC(C3=CN=C(NC4=CC=CC=C4)S3)=NO2 | -7.232 |  |
| 125 | C125 | OC(C([N+]([O-])=O)=CC(OC)=C1)=C1C2=CC(C3=CN=C(NC4=CC=CC=C4)S3)=NO2 | -6.945 |  |
| 126 | C126 | COC1=CC=C([N+]([O-])=O)C(C2=CC(C3=CN=C(NC4=CC=CC=C4)S3)=NO2)=C1[N+]([O-])=O | -8.666 |  |
| 127 | C127 | ClC1=CC(Cl)=CC(C2=CC(C3=CN=C(NC4=CC=CC=C4)S3)=NO2)=C1Cl | -7.938 |  |
| 128 | C128 | CC1=C(C2=NOC(C3=CC(OC)=C(F)C=C3)=C2)SC(NC4=CC=CC=C4)=N1 | -7.102 |  |
| 129 | C129 | IC1=C(OC)C=CC(C2=CC(C3=CN=C(NC4=CC=CC=C4)S3)=NO2)=C1 | -7.617 |  |
| 130 | C130 | ClC1=CC=C(OC)C(Cl)=C1C2=CC(C3=CN=C(NC4=CC=CC=C4)S3)=NO2 | -7.267 |  |
| 131 | C131 | COC(C=C1)=C(OC(C)C)C=C1C2=CC(C3=CN=C(NC4=CC=CC=C4)S3)=NO2 | -10.207 |  |
| 132 | C132 | COC1=CC=CC(C2=CC(C3=CN=C(NC4=CC=CC=C4)S3)=NO2)=C1OCC5=CC=CC(Cl)=C5 | -7.528 |  |
| 133 | C133 | COC(C=C1)=C(OCC)C=C1C2=CC(C3=CN=C(NC4=CC=CC=C4)S3)=NO2 | -8.607 |  |
| 134 | C134 | CC1=C(C2=NOC(C3=CC(F)=C(O)C(OC)=C3)=C2)SC(NC4=CC=CC=C4)=N1 | -7.449 |  |
| 135 | C135 | CC1=C(C2=NOC(C3=CC(F)=C(OC)C=C3)=C2)SC(NC4=CC=CC=C4)=N1 | -7.436 |  |
| 136 | C136 | CC1=C(C2=NOC(C3=CC(Cl)=C(OC)C=C3)=C2)SC(NC4=CC=CC=C4)=N1 | -8.171 |  |
| 137 | C137 | CC1=C(C2=NOC(C3=C(Br)C(O)=C(OC)C=C3)=C2)SC(NC4=CC=CC=C4)=N1 | -6.493 |  |
| 138 | C138 | CC1=C(C2=NOC(C3=CC(OC)=C(OCC4=CC=CC=C4)C=C3)=C2)SC(NC5=CC=CC=C5)=N1 | -8.284 |  |
| 139 | C139 | CC1=C(C2=NOC(C3=CC(Br)=C(OC(C)C)C(OC)=C3)=C2)SC(NC4=CC=CC=C4)=N1 | -11.114 |  |
| 140 | C140 | CC1=C(C2=NOC(C3=C(OCC4=CC(Cl)=C(Cl)C=C4)C(OC)=CC=C3)=C2)SC(NC5=CC=CC=C5)=N1 | -7.385 |  |
| 141 | C141 | CC1=C(C2=NOC(C3=CC=CC=C3)=C2)SC(NC4=CC=C(Cl)C=C4)=N1 | -8.741 |  |
| 142 | C142 | CC1=C(C2=NOC(C3=C(O)C=CC=C3)=C2)SC(NC4=CC=C(Cl)C=C4)=N1 | -8.656 |  |
| 143 | C143 | CC1=C(C2=NOC(C3=C(Cl)C=CC=C3)=C2)SC(NC4=CC=C(Cl)C=C4)=N1 | -8.511 |  |
| 144 | C144 | CC1=C(C2=NOC(C3=CC(Cl)=CC=C3)=C2)SC(NC4=CC=C(Cl)C=C4)=N1 | -8.213 |  |
| 145 | C145 | CC1=C(C2=NOC(C3=CC=C(Cl)C=C3)=C2)SC(NC4=CC=C(Cl)C=C4)=N1 | -8.874 |  |
| 146 | C146 | CC1=C(C2=NOC(C3=C(Cl)C=C(Cl)C=C3)=C2)SC(NC4=CC=C(Cl)C=C4)=N1 | -8.312 |  |
| 147 | C147 | ClC1=CC=CC(Cl)=C1C2=CC(C3=CN=C(NC4=CC=C(Cl)C=C4)S3)=NO2 | -8.400 |  |
| 148 | C148 | ClC(C(Cl)=CC=C1)=C1C2=CC(C3=CN=C(NC4=CC=C(Cl)C=C4)S3)=NO2 | -8.638 |  |
| 149 | C149 | ClC1=CC(Cl)=CC(C2=CC(C3=CN=C(NC4=CC=C(Cl)C=C4)S3)=NO2)=C1 | -7.931 |  |
| 150 | C150 | ClC1=C(Cl)C=CC(C2=CC(C3=CN=C(NC4=CC=C(Cl)C=C4)S3)=NO2)=C1 | -8.117 |  |
| 151 | C151 | ClC(C=C1)=CC=C1NC2=NC=C(C3=NOC(C4=CC([N+]([O-])=O)=CC=C4)=C3)S2 | -7.568 |  |
| 152 | C152 | ClC(C=C1)=CC=C1NC2=NC=C(C3=NOC(C4=CC=C([N+]([O-])=O)C=C4)=C3)S2 | -8.349 |  |
| 153 | C153 | ClC(C=C1)=CC=C1NC2=NC=C(C3=NOC(C4=C([N+]([O-])=O)C=CC=C4)=C3)S2 | -7.719 |  |
| 154 | C154 | ClC(C=C1)=CC=C1NC2=NC=C(C3=NOC(C4=C([N+]([O-])=O)C=C([N+]([O-])=O)C=C4)=C3)S2 | -7.724 |  |
| 155 | C155 | ClC(C=C1)=CC=C1NC2=NC=C(C3=NOC(C4=C([N+]([O-])=O)C=CC=C4[N+]([O-])=O)=C3)S2 | -7.724 |  |
| 156 | C156 | ClC(C=C1)=CC=C1NC2=NC=C(C3=NOC(C4=CC([N+]([O-])=O)=CC([N+]([O-])=O)=C4)=C3)S2 | -6.311 |  |
| 157 | C157 | CC1=C(C)C(C)=CC(C2=CC(C3=CN=C(NC4=CC=C(Cl)C=C4)S3)=NO2)=C1 | -7.807 |  |
| 158 | C158 | ClC(C=C1)=CC=C1NC2=NC=C(C3=NOC(C4=C(OC)C=CC=C4)=C3)S2 | -7.983 |  |
| 159 | C159 | ClC(C=C1)=CC=C1NC2=NC=C(C3=NOC(C4=CC=C(OC)C=C4)=C3)S2 | -7.518 |  |
| 160 | C160 | ClC(C=C1)=CC=C1NC2=NC=C(C3=NOC(C4=C(OC)C=CC(OC)=C4)=C3)S2 | -7.005 |  |
| 161 | C161 | ClC(C=C1)=CC=C1NC2=NC=C(C3=NOC(C4=CC(OC)=C(OC)C=C4)=C3)S2 | -5.488 |  |
| 162 | C162 | CN(C)C(C=C1)=CC=C1C2=CC(C3=CN=C(NC4=CC=C(Cl)C=C4)S3)=NO2 | -8.027 |  |
| 163 | C163 | FC(C=C1)=CC=C1C2=CC(C3=CN=C(NC4=CC=C(Cl)C=C4)S3)=NO2 | -7.981 |  |
| 164 | C164 | BrC(C=C1)=CC=C1C2=CC(C3=CN=C(NC4=CC=C(Cl)C=C4)S3)=NO2 | -8.367 |  |
| 165 | C165 | CC1=C(C2=NOC(C3=CC=C(O)C=C3)=C2)SC(NC4=CC=C(Cl)C=C4)=N1 | -6.921 |  |
| 166 | C166 | ClC(C=C1)=CC=C1NC2=NC=C(C3=NOC(C4=CC(OC)=C(OC)C(OC)=C4)=C3)S2 | -8.716 |  |
| 167 | C167 | CC1=C(C2=NOC(C3=CC(OC)=C(O)C=C3)=C2)SC(NC4=CC=C(Cl)C=C4)=N1 | -10.172 |  |
| 168 | C168 | CC1=C(C2=NOC(C3=C(Cl)C=CC(C(F)(F)F)=C3)=C2)SC(NC4=CC=C(Cl)C=C4)=N1 | -8.982 |  |
| 169 | C169 | CC1=C(C2=NOC(C3=CC=C(C(F)(F)F)C=C3)=C2)SC(NC4=CC=C(Cl)C=C4)=N1 | -8.741 |  |
| 170 | C170 | CC1=C(C2=NOC(C3=CC=C(C(F)(F)F)C=C3)=C2)SC(NC4=CC=C(Cl)C=C4)=N1 | -8.701 |  |
| 171 | C171 | CC1=C(C2=NOC(C3=C(Cl)C=C(N)C=C3)=C2)SC(NC4=CC=C(Cl)C=C4)=N1 | -9.311 |  |
| 172 | C172 | CC1=C(C2=NOC(C3=CC(OC)=C(O)C([N+]([O-])=O)=C3)=C2)SC(NC4=CC=C(Cl)C=C4)=N1 | -8.800 |  |
| 173 | C173 | BrC(C=C(O)C(OC)=C1)=C1C2=CC(C3=CN=C(NC4=CC=C(Cl)C=C4)S3)=NO2 | -8.973 |  |
| 174 | C174 | CC1=C(C2=NOC(C3=CC(OC)=C(O)C(Br)=C3)=C2)SC(NC4=CC=C(Cl)C=C4)=N1 | -8.858 |  |
| 175 | C175 | OC(C(OCC)=C1)=C(Br)C=C1C2=CC(C3=CN=C(NC4=CC=C(Cl)C=C4)S3)=NO2 | -9.029 |  |
| 176 | C176 | CC1=C(C2=NOC(C3=CC(OC)=C(O)C(I)=C3)=C2)SC(NC4=CC=C(Cl)C=C4)=N1 | -8.869 |  |
| 177 | C177 | BrC1=C(OCCCCC)C(OC)=CC(C2=CC(C3=CN=C(NC4=CC=C(Cl)C=C4)S3)=NO2)=C1 | -8.869 |  |
| 178 | C178 | COC(C=C1)=C(OCC2=CC=CC=C2)C(Br)=C1C3=CC(C4=CN=C(NC5=CC=C(Cl)C=C5)S4)=NO3 | -8.957 |  |
| 179 | C179 | CC1=C(C2=NOC(C3=CC=C(OCC4=CC=CC=C4)C=C3)=C2)SC(NC5=CC=C(Cl)C=C5)=N1 | -5.923 |  |
| 180 | C180 | FC1=C(OC(F)(F)F)C=CC(C2=CC(C3=CN=C(NC4=CC=C(Cl)C=C4)S3)=NO2)=C1 | -8.168 |  |
| 181 | C181 | CC1=C(C2=NOC(C3=C(F)C=C(N4CCCOC4)C=C3)=C2)SC(NC5=CC=C(Cl)C=C5)=N1 | -8.048 |  |
| 182 | C182 | ClC(C=C1)=CC=C1NC2=NC=C(C3=NOC(C4=CC(F)=C(S(C)(=O)=O)C=C4)=C3)S2 | -7.050 |  |
| 183 | C183 | ClC(C=C1)=CC=C1NC2=NC=C(C3=NOC(C4=CC(F)=CC=C4N5N=CC=C5)=C3)S2 | -5.019 |  |
| 184 | C184 | CC1=C(C2=NOC(C3=CC=CC=C3C4=CN=CN=C4)=C2)SC(NC5=CC=C(Cl)C=C5)=N1 | -8.084 |  |
| 185 | C185 | CC1=C(C2=NOC(C3=C(C)C=C(N4CCCC4)C(F)=C3)=C2)SC(NC5=CC=C(Cl)C=C5)=N1 | -10.450 |  |
| 186 | C186 | ClC(C=C1)=CC=C1NC2=NC=C(C3=NOC(C4=CC(OC)=CC=C4)=C3)S2 | -8.462 |  |
| 187 | C187 | CC1=C(C2=NOC(C3=C(O)C=CC([N+]([O-])=O)=C3)=C2)SC(NC4=CC=C(Cl)C=C4)=N1 | -7.069 |  |
| 188 | C188 | CC1=C(C2=NOC(C3=C([N+]([O-])=O)C=CC(O)=C3)=C2)SC(NC4=CC=C(Cl)C=C4)=N1 | -8.381 |  |
| 189 | C189 | CC1=C(C2=NOC(C3=C([N+]([O-])=O)C=CC(Cl)=C3)=C2)SC(NC4=CC=C(Cl)C=C4)=N1 | -8.619 |  |
| 190 | C190 | CC1=C(C2=NOC(C3=C(OC)C=C([N+]([O-])=O)C=C3)=C2)SC(NC4=CC=C(Cl)C=C4)=N1 | -8.974 |  |
| 191 | C191 | CC1=C(C2=NOC(C3=C([N+]([O-])=O)C(OC)=CC=C3)=C2)SC(NC4=CC=C(Cl)C=C4)=N1 | -8.090 |  |
| 192 | C192 | ClC(C=CC([N+]([O-])=O)=C1)=C1C2=CC(C3=CN=C(NC4=CC=C(Cl)C=C4)S3)=NO2 | -8.129 |  |
| 193 | C193 | COC1=CC(C2=CC(C3=CN=C(NC4=CC=C(Cl)C=C4)S3)=NO2)=C([N+]([O-])=O)C=C1 | -8.176 |  |
| 194 | C194 | OC(C([N+]([O-])=O)=CC=C1)=C1C2=CC(C3=CN=C(NC4=CC=C(Cl)C=C4)S3)=NO2 | -8.501 |  |
| 195 | C195 | OC(C([N+]([O-])=O)=CC(OC)=C1)=C1C2=CC(C3=CN=C(NC4=CC=C(Cl)C=C4)S3)=NO2 | -8.501 |  |
| 196 | C196 | COC1=CC=C([N+]([O-])=O)C(C2=CC(C3=CN=C(NC4=CC=C(Cl)C=C4)S3)=NO2)=C1[N+]([O-])=O | -8.190 |  |
| 197 | C197 | ClC1=CC(Cl)=CC(C2=CC(C3=CN=C(NC4=CC=C(Cl)C=C4)S3)=NO2)=C1Cl | -7.788 |  |
| 198 | C198 | CC1=C(C2=NOC(C3=CC(OC)=C(F)C=C3)=C2)SC(NC4=CC=C(Cl)C=C4)=N1 | -9.120 |  |
| 199 | C199 | IC1=C(OC)C=CC(C2=CC(C3=CN=C(NC4=CC=C(Cl)C=C4)S3)=NO2)=C1 | -8.400 |  |
| 200 | C200 | ClC1=CC=C(OC)C(Cl)=C1C2=CC(C3=CN=C(NC4=CC=C(Cl)C=C4)S3)=NO2 | -7.629 |  |
| 201 | C201 | COC(C=C1)=C(OC(C)C)C=C1C2=CC(C3=CN=C(NC4=CC=C(Cl)C=C4)S3)=NO2 | -8.763 |  |
| 202 | C202 | ClC(C=C1)=CC=C1NC2=NC=C(C3=NOC(C4=C(OCC5=CC=CC(Cl)=C5)C(OC)=CC=C4)=C3)S2 | -5.586 |  |
| 203 | C203 | ClC(C=C1)=CC=C1NC2=NC=C(C3=NOC(C4=CC(OCC)=C(OC)C=C4)=C3)S2 | -10.017 |  |
| 204 | C204 | CC1=C(C2=NOC(C3=CC(F)=C(O)C(OC)=C3)=C2)SC(NC4=CC=C(Cl)C=C4)=N1 | -7.597 |  |
| 205 | C205 | CC1=C(C2=NOC(C3=CC(F)=C(OC)C=C3)=C2)SC(NC4=CC=C(Cl)C=C4)=N1 | -9.175 |  |
| 206 | C206 | CC1=C(C2=NOC(C3=CC(Cl)=C(OC)C=C3)=C2)SC(NC4=CC=C(Cl)C=C4)=N1 | -8.155 |  |
| 207 | C207 | CC1=C(C2=NOC(C3=C(Br)C(O)=C(OC)C=C3)=C2)SC(NC4=CC=C(Cl)C=C4)=N1 | -8.072 |  |
| 208 | C208 | CC1=C(C2=NOC(C3=CC(OC)=C(OCC4=CC=CC=C4)C=C3)=C2)SC(NC5=CC=C(Cl)C=C5)=N1 | -9.093 |  |
| 209 | C209 | CC1=C(C2=NOC(C3=CC(Br)=C(OC(C)C)C(OC)=C3)=C2)SC(NC4=CC=C(Cl)C=C4)=N1 | -5.290 |  |
| 210 | C210 | CC1=C(C2=NOC(C3=C(OCC4=CC(Cl)=C(Cl)C=C4)C(OC)=CC=C3)=C2)SC(NC5=CC=C(Cl)C=C5)=N1 | -9.386 |  |
| 211 | pio | CCC1=CN=C(C=C1)CCOC2=CC=C(C=C2)CC3C(=O)NC(=O)S3 |  |  |

**Table 2: Residue interaction of the top nine compounds, pioglitazone and the Co-crystal (Q-35) with 5GTN receptor**

| Compound name | Binding affinity (kcal/mol) | Hydrogen bonding | Residue binding through other interaction |
| --- | --- | --- | --- |
| C139 | -11.114 |  | PHE264,ILE262,ILE341,VAL339,LEU340,LEU353,MET334,LEU255,PHE368,MET364,PHE363,LEU330,TYR327,ILE326,LEU453,LEU469,TYR473,CYS285,MET348,PHE282,ILE281(hydrophobic interaction) |
|  |  |  | HIE449,SER289,SER342,HIS323,GLN286(polar interaction) |
|  |  |  | LYS263,ARG288,LYS367,ARG280(+ve charged) |
|  |  |  | GLU259(-ve charrged) |
|  |  |  | HIE449,PHE264(Pi-Pi stacking) |
| C184 | -10.450 | SER342 | LEU255,LEU333,MET348,LEU330,TYR327,ILE326,LEU453,TYR473,LEU469,PHE287,CYS285,ILE281,ILE341,VAL339,PHE264,ILE262(hydrophobic interaction) |
|  |  |  | SER342,GLN286,SER289,HIE449,HIS323(polar interaction) |
|  |  |  | ARG288,LYS263(+ve charged) |
|  |  |  | GLU259(-ve charrged) |
| C107 | -10.273 | LYS263 | PHE363,MET364,MET334,PHE368,LEU353,MET348,VAL339,ILE341,PHE264,PRO227,PHE287,CYS285,PHE282,LEU330,TYR327,ILE326(hydrophobic interaction) |
|  |  |  | SER289,HIS323,HIE449,SER342(polar interaction) |
|  |  |  | ARG288,LYS263,LYS367(+ve charged) |
|  |  |  | GLU343,GLU291,GLU295(-ve charrged) |
|  |  |  | LYS263(Pi-cation) |
|  |  |  | HIE449(Pi-Pi stacking) |
| C131 | -10.207 |  | LEU255,ILE262,PHE264,ILE281,PHE282,CYS285,VAL339,ILE341,LEU469,TYR473,LEU453,ILE326,TYR327,LEU330,PHE363,MET364,MET334,LEU353,PHE368,MET348(hydrophobic interaction) |
|  |  |  | HIS323,HIE449,SER342,SER289,GLN286(polar interaction) |
|  |  |  | LYS367,ARG288,ARG280,LYS263(+ve charged) |
|  |  |  | GLU259(-ve charrged) |
|  |  |  | HIS323(halogen bond) |
|  |  |  | HIE449(Pi-Pi stacking) |
| C167 | -10.172 |  | ILE262,PHE264,ILE281,PHE282,CYS285,PHE287,ILE281,VAL339,ILE341,LEU453,LEU469,TYR473,ILE326,TYR327,LEU330,LEU353,MET334,MET348,LEU255(hydrophobic interaction) |
|  |  |  | SER342,SER289,HIS323,HIE449(polar interaction) |
|  |  |  | ARG280,LYS263,ARG288,LYS367(+ve charged) |
|  |  |  | GLU259(-ve charrged) |
|  |  |  | SER342(halogen bond) |
|  |  |  | HIE449(Pi-Pi stacking) |
| C113 | -10.163 | SER342 | ILE281,CYS285,PHE287,ILE341,VAL339,PHE264,ILE262,LEU333,LEU330,TYR327,ILE326,TYR473,LEU453,MET348,LEU255(hydrophobic interaction) |
|  |  |  | HIS323,HIE449,SER289,SER342,GLN286(polar interaction) |
|  |  |  | ARG288,LYS263(+ve charged) |
|  |  |  | GLU259(-ve charrged) |
| C202 | -10.017 |  | LEU330,TYR327,ILE326,TYR473,LEU453,LEU255,PHE264,ILE262,ILE281,PHE282,CYS285,MET348VAL339,ILE341,LEU353,PHE368,MET334,MET364,PHE363(hydrophobic interaction) |
|  |  |  | HIS323,HIE449,SER342,SER289(polar interaction) |
|  |  |  | LYS367,ARG288,ARG280,LYS263(+ve charged) |
|  |  |  | GLU259(-ve charged) |
|  |  |  | HIE449,PHE264(Pi-Pi stacking) |
| C37 | -9.998 | LYS263 | LEU330,TYR327,ILE326,PHE282,CYS285,PHE287,ILE262,PHE264,ILE341,VAL339,LEU353,PHE368,MET334,MET364,PHE363(hydrophobic interaction) |
|  |  |  | HIE449,SER289,SER342(polar interaction) |
|  |  |  | LYS263,ARG288,LYS367(+ve charged) |
|  |  |  | GLU291,GLU343(-ve charged) |
| C29 | -9.970 | LYS263 | ILE262,PHE264,PHE287,CYS285,ILE281,MET348,LEU353,PHE363,MET364,MET334,PHE368,LEU330,VAL339,ILE341(hydrophobic interaction) |
|  |  |  | SER342(polar interaction) |
|  |  |  | ARG288,LYS263,LYS367(+ve charged) |
|  |  |  | GLU343,GLU293(-ve charged) |
| Pioglitazone (std) | -7.630 |  | PHE264,ILE262,LEU255,ILE281,MET348,ILE341,VAL339,CYS285,LEU353,PHE363,MET334,MET364,LEU330,TYR327,ILE326(hydrophobic interaction) |
|  |  |  | HIE449,SER342(polar interaction) |
|  |  |  | LYS367,ARG288,ARG280(+ve charged) |
|  |  |  | GLU259(-ve charged) |
| Co crystal  Q-35 | -10.33 | HIS 449, TYR473 | LEU 255, ILE 262, PHE 264, ILE 281, PHE282, CYS285, PHE 287, ALA292, MET 334, LEU 333, LEU 330, TYR327, ILE 326, LEU 340, ILE341, VAL 339, MET 348, PHE 368, LEU 353, MET 364, PHE363, LEU 469, TYR 473, LEU 453, LEU 465 (HYDROPHOBIC INTERACTION) |
|  |  |  | GLN 286, SER 289, HIS 323, SER 342, HIS 449 (POLAR INTERATION) |
|  |  |  | LYS 263, ARG 280, ARG288, LYS 367 (POSITIVE CHARGED INTERACTION) |
|  |  |  | HIS 449 (Pi-Pi interaction) |
